# KRAS mutation increases histone H3 lysine 9 lactylation (H3K9la) to promote colorectal cancer progression by facilitating cholesterol transporter GRAMD1A expression

**DOI:** 10.1038/s41418-025-01533-4

**Published:** 2025-07-24

**Authors:** Chi Zhang, Runfeng Yu, Senmao Li, Ming Yuan, Tuo Hu, Jiaqi Liu, Haoxian Ke, Shubiao Ye, Jihye Yun, Junfeng Huang, Guanzhan Liang, Shaopeng Chen, Xianrui Wu, Ping Lan

**Affiliations:** 1 Department of General Surgery (Colorectal Surgery), The Sixth Affiliated Hospital, Sun Yat-sen University, Guangzhou, Guangdong China; 2 Guangdong Provincial Key Laboratory of Colorectal and Pelvic Floor Diseases, The Sixth Affiliated Hospital, Sun Yat-sen University, Guangzhou, Guangdong China; 3 Biomedical Innovation Center, The Sixth Affiliated Hospital, Sun Yat-sen University, Guangzhou, Guangdong China; 4 Department of Gastrointestinal Surgery, Sun Yat-sen Memorial Hospital, Sun Yat-sen University, Guangzhou, Guangdong China; 5 Department of Clinical Laboratory, The Sixth Affiliated Hospital, Sun Yat-sen University, Guangzhou, Guangdong China; 6 Department of Genetics, MD Anderson Cancer Center, The University of Texas, Houston, TX USA; 7 State Key Laboratory of Oncology in South China, Guangzhou, Guangdong China

**Keywords:** Genetics research, Cancer genetics

## Abstract

Histone lactylation is a novel epigenetic modification derived from lactate, but its role and mechanism in KRAS mutant colorectal cancer (CRC) progression remains to be fully elucidated. In this study, we first showed that mutant KRAS increased H3 lysine 9 lactylation (H3K9la) to promote CRC progression. We found that KRAS-mutant CRC tissues and cell lines exhibited higher lactylation and H3K9la levels compared to KRAS wild-type counterparts, driven by increased intracellular lactate. Elevated lactylation and H3K9la levels were associated with poor prognosis and advanced clinical stages. Inhibition of lactylation and H3K9la suppressed proliferation and migration of CRC cells. Mechanistically, mutant KRAS upregulated GRAMD1A expression by elevating H3K9la levels to increase chromatin accessibility. And increased GRAMD1A facilitated cholesterol metabolism to promote CRC growth and metastasis. Targeted inhibition of H3K9la or GRAMD1A reduced tumor growth in CRC patient-derived xenografts (PDX) models. Our study uncovered the critical role of H3K9la as a novel epigenetic modification in KRAS mutant CRC progression, suggesting H3K9la and its downstream gene GRAMD1A as promising targets for therapeutic intervention in KRAS mutant CRC and potential biomarkers for the prognosis of CRC patients.

## Introduction

Colorectal cancer (CRC) is the third most common malignancy worldwide, accounting for nearly 8.5% of all cancer deaths [1]. Despite advances in treatment, recurrence and distant metastasis remain primary challenges leading to mortality among CRC patients [2]. Current therapies for metastatic CRC offer limited improvements in patient outcomes [3]. Therefore, understanding the molecular mechanisms underlying CRC pathogenesis is crucial for developing more effective therapeutic strategies and improving clinical outcomes.

The KRAS oncogene is mutated in approximately 40% of CRC patients and typically associated with resistance to receptor tyrosine kinase inhibitors, distant metastasis, poor tumor differentiation and reduced survival rate [4]. Genetically engineered CRC mouse models have elucidated the role of KRAS in CRC pathogenesis, showing that conditional expression of mutant KRAS alleles promotes tumor progression [5]. Moreover, emerging evidence suggests that KRAS mutations induce metabolic reprogramming in CRC cells, with enhanced glycolysis being one of the most evident and critical alterations, leading to increased lactate production [6]. Although research on metabolic changes has improved our understanding of KRAS-mutation-driven CRC, the precise mechanism by which metabolic reprogramming promotes and coordinates KRAS-mutant CRC progression remains to be fully elucidated.

Histone lactylation is a recently discovered epigenetic modification derived from lactate, which modulates histones by adding lactyl groups from lactate to their lysine residues. To date, 28 potential lactylation sites have been identified on core histones [7]. Among these, histone H3 lysine 18 lactylation (H3K18la) is the most extensively studied, which has been shown to stimulates gene transcription to regulate numerous cancer biological processes, including tumorigenesis [8, 9], cancer progression [10–12], and tumor immune escape [13]. However, the role of other lactylation sites remain to be further explored.

In this study, we revealed that lactylation and histone H3 lysine 9 lactylation (H3K9la) levels were elevated in KRAS mutant CRC due to increased intracellular lactate, and associated with poor prognosis and advanced clinical stages. Inhibiting lactylation and H3K9la efficiently suppressed proliferation and migration of KRAS mutant CRC cells. Mechanistically, mutant KRAS elevated H3K9la levels at the GRAMD1A promoter region, enhancing chromatin accessibility and thereby promoting GRAMD1A expression. GRAMD1A, a cholesterol transporter, facilitated cholesterol metabolism, driving CRC growth and metastasis. Targeted inhibition of H3K9la or GRAMD1A suppressed the tumor growth in CRC patient-derived xenografts (PDX) models. Our findings suggested that inhibiting H3K9la or GRAMD1A could represent a novel strategy for treating KRAS mutant CRC.

## Materials and methods

Additional methods are provided in the Supplementary information.

### Patients and specimens

Formalin-fixed, paraffin-embedded CRC tissues, normal colon tissues, and liquid nitrogen-stored CRC and normal colon tissues were collected from the Sixth Affiliated Hospital of Sun Yat-sen University. The use of clinical samples, with informed patient consent, was approved by the Institutional Review Board of the Sixth Affiliated Hospital of Sun Yat-sen University (2023ZSLYEC-447).

### Statistical analysis

The results, derived from a minimum of three independent repetitions, are presented as mean ± standard deviation (SD). All data analyses were conducted by GraphPad Prism software (La Jolla, CA, USA) or R software. For continuous variables with approximately normal distribution and independent samples, two-tailed Student’s t-test or one-way analysis of variance (ANOVA) was used to compare the means among groups. Data normality was assessed using the Shapiro-Wilk test, and homogeneity of variance was evaluated using F-test. If the assumption of equal variances was violated, Welch’s t-test or Welch’s ANOVA was applied. For discrete or non-normally distributed data, statistical significance was calculated using Mann-Whitney U test or Kruskal–Wallis test. Survival analysis was performed using the Kaplan–Meier and Cox analysis. Refer to the individual figure legends for precise details about sample sizes and statistical approaches employed for each finding.

## Results

### KRAS mutant colorectal cancer (CRC) exhibited elevated lactylation which was associated with poor patient survival

**Fig. 1:**
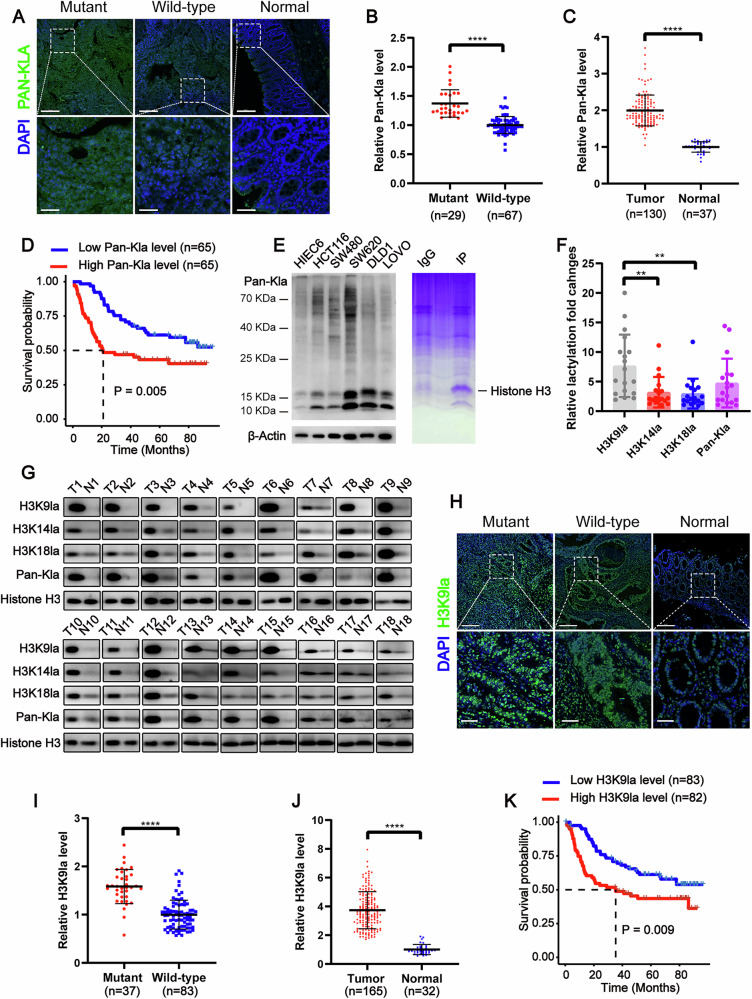
KRAS-mutant colorectal cancer (CRC) exhibited elevated lactylation which was associated with poor patient survival. **A** Immunofluorescence staining of lactylation levels in KRAS mutant and wild-type CRC tissues and normal colon tissues. Scale bar: up panel, 200 μm; down panel, 50 μm. **B** Statistical immunofluorescence results of lactylation levels in KRAS mutant and wild-type CRC tissues. **C** Statistical immunofluorescence results of lactylation levels in CRC tissues and normal colon tissues. **D** Kaplan–Meier survival curves contrasting difference between CRC patients with low and high lactylation levels. **E** Left, western blot analysis showing lactylation level in normal intestinal epithelial cell line (HIEC6) and CRC cell lines (HCT116, SW480, SW620, DLD1, LOVO); Right, Coomassie blue staining -mass spectrometry (MS) of lactylated proteins in HCT116 cells. **F**, **G** lactylation level of different histone H3 lysine residues. **F** Statistical results of lactylation fold changes between tumor and normal tissues of histone H3 lysine 9 lactylation (H3K9la), histone H3 lysine 14 lactylation (H3K14la) and histone H3 lysine 18 lactylation (H3K18la) in CRC tissues. **G** Western blot analysis showing H3K9la, H3K14la and H3K18la levels in CRC tissues and normal colon tissues. **H** Immunofluorescence staining of H3K9la levels in KRAS mutant and wild-type CRC tissues and normal colon tissues. Scale bar: 200 μm. **I** Statistical results of H3K9la levels in KRAS mutant and wild-type CRC tissues. **J** Statistical results of H3K9la levels in CRC tissues and normal colon tissues. **K** Kaplan-Meier survival curves contrasting difference between CRC patients with low and high H3K9la levels. Values are presented as mean ± SD. **p < 0.01, ****p < 0.0001, determined by Mann–Whitney U test (**B**, **C**, **I**, **J**), Kruskal–Wallis test (**F**) and Log-rank test (**D**, **K**).

To evaluate the lysine lactylation levels in CRC and their conceivable clinical significance, we performed immunofluorescence staining on 130 CRC tissues and 37 normal colon tissues from patients. The result showed significantly higher lactylation levels (Pan-Kla) in KRAS mutant CRC tissues compared to KRAS wild-type CRC tissues, and in CRC tissues compared to normal colon tissues (Fig. 1A–C). Moreover, elevated lactylation levels were associated with poor survival and advanced clinical stages in CRC patients and could serve as an independent predictor of survival. (Figs. 1D and S1A, B). Consistently, western blot analysis revealed increased lactylation in CRC cell lines compared to normal intestinal epithelial cell line (HIEC-6), with the predominant band near 18 kDa (Fig. 1E, left). To further determine the proteins in this predominant band, we conducted Coomassie blue staining-mass spectrometry (MS) after immunoprecipitation of lactylated proteins, identifying histone H3 as the primary protein (Fig. 1E, right). Given that histone H3 has multiple potential lysine lactylation sites, we assessed lactylation levels at different lysine residues using available specific antibodies. Western blot analysis of paired tumor and normal colon tissue samples from 18 CRC patients revealed that histone H3 lysine 9 (H3K9) showed significantly higher lactylation fold changes between tumor and normal tissues compared to histone H3 lysine 14 (H3K14) and histone H3 lysine 18 (H3K18) (Fig. 1F, G). Based on these findings, we focused on histone H3 lysine 9 lactylation (H3K9la). To evaluate the H3K9la levels in CRC and their clinical significance, we performed immunofluorescence staining on 165 CRC tissues and 32 normal colon tissues from patients. Our results showed significantly higher H3K9la levels in KRAS-mutant CRC tissues compared to KRAS wild-type CRC tissues, and in CRC tissues compared to normal colon tissues (Fig. 1H–J). Higher H3K9la levels was associated with poorer survival and more advanced clinical stages in CRC patients and could serve as an independent predictor of survival (Figs. 1K and S1C, D). These findings suggested that increased lactylation and H3K9la were positively correlated with KRAS mutations and poor prognosis in CRC patients.

### KRAS mutations elevated H3K9la by increasing lactate production

**Fig. 2:**
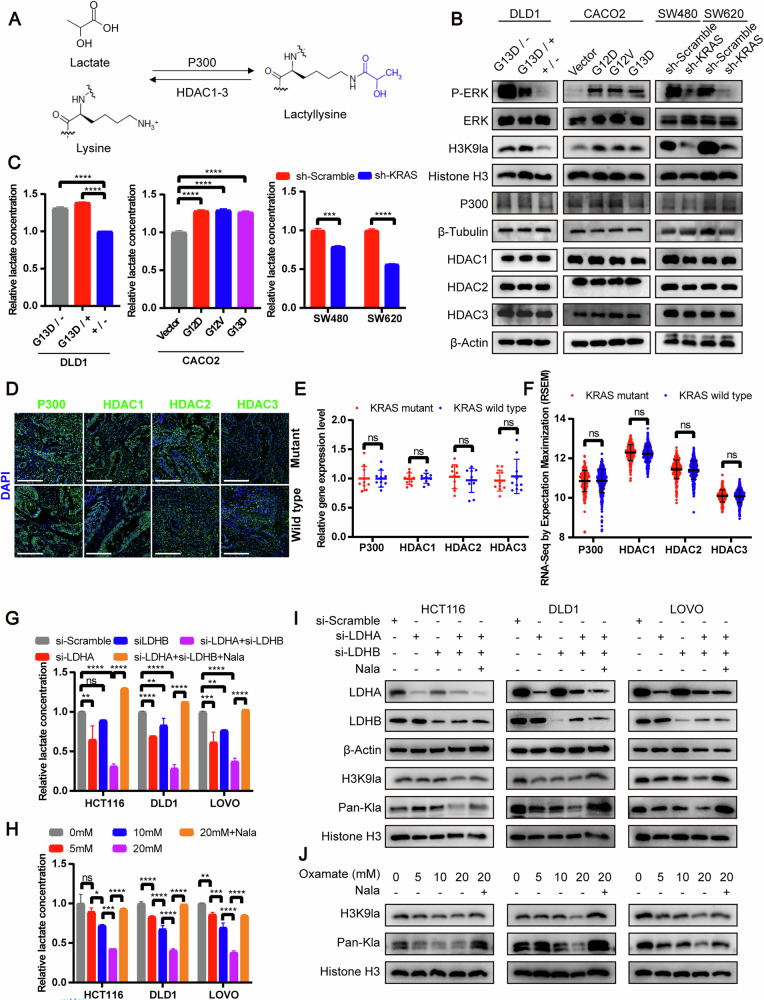
KRAS mutation elevated H3K9la by increasing lactate production. **A** Schematic diagram of histone lactylation process. **B** Western blot of H3K9la, P300, HDAC1, HDAC2 and HDAC3 levels and **C** Intracellular lactate levels measured by a lactate colorimetric kit, in DLD1 cell lines harboring wild-type KRAS (+/−), G13D mutant KRAS (G13D/−) or both alleles (G13D/+) of KRAS (left), CACO2 cells stably transfected with vector and cDNA encoding the KRAS^G12D^, KRAS^G12V^ and KRAS^G13D^ mutations (middle), and SW480 and SW620 cells transfected with KRAS-specific shRNA (right). *n*  =  3. **D** Immunofluorescence staining of P300, HDAC1, HDAC2 and HDAC3 expressions in KRAS mutant and wild-type CRC tissues. Scale bar: 200 μm. **E** Statistical immunofluorescence results of P300, HDAC1, HDAC2 and HDAC3 expressions in KRAS mutant and wild-type CRC tissues. **F** Statistical results of P300, HDAC1, HDAC2 and HDAC3 expressions in KRAS mutant and wild-type CRC tissues in TCGA datasets of CRC. Intracellular lactate levels of HCT116, DLD1 and LOVO cells (**G**) cultured with or without 10 mM Nala for 24 h after silencing LDHA, LDHB or both and (**H**) cultured in different concentration of oxamate with or without 10 mM Nala for 24 h by a lactate colorimetric kit. *n*  =  3. H3K9la and lactylation levels of HCT116, DLD1 and LOVO cells (**I**) cultured with or without 10 mM Nala for 24 h after silencing LDHA, LDHB or both and (**J**) cultured in different concentration of oxamate with or without 10 mM Nala for 24 h by Western blot. Values are presented as mean ± SD. **p < 0.01, *** *p* < 0.001, **** *p* < 0.0001, ns *p* > 0.05, determined by one-way ANOVA (**C**, **G**, **H**) and two-tailed Student’s t-test (**C**, **E**, **F**).

To understand the mechanism by which KRAS mutations increased H3K9la, we assessed the levels of lactate, the substrate for lactylation, as well as the expressions of the lactylation writer P300 [7] and lactylation erasers histone deacetylase (HDAC) 1, 2 and 3 [14] (Fig. 2A). In isogenic DLD1 cell lines harboring wild-type KRAS (+/−), G13D mutant KRAS (G13D/−) or both alleles (G13D/+) of KRAS [15], mutant KRAS elevated the H3K9la levels and increased the intracellular lactate concentration, but did not affect the expression of lactylation writer P300 and lactylation erasers histone deacetylase (HDAC) 1, 2 and 3 significantly (Fig. 2B, C). We also stably transfected cDNA encoding the KRAS^G12D^, KRAS^G12v^ and KRAS^G13D^ mutations into KRAS wild-type CACO2 cells. Induction of KRAS mutations elevated the H3K9la and intracellular lactate levels, but had no obvious influence on P300 and HDAC 1, 2 and 3 expressions (Fig. 2B, C). Moreover, disruption of KRAS signaling using KRAS-specific short hairpin RNA (shRNA) in KRAS mutant SW480 and SW620 cells effectively reduced H3K9la and intracellular lactate levels without affecting P300 and HDAC1, 2, and 3 expressions remarkably (Fig. 2B, C). Immunofluorescence staining (Fig. 2D, E) and transcriptome data in The Cancer Genome Atlas (TCGA) database (Fig. 2F) also revealed no significant difference in P300 and HDAC1, 2, and 3 expressions between KRAS mutant and wild-type CRC tissues. These findings suggested that mutant KRAS increased intracellular lactate levels and elevated H3K9la levels, without significantly altering the expression of lactylation writer P300 or erasers HDAC1-3. To further investigate the role of lactate in regulating H3K9la, we inhibited lactate production using the lactate dehydrogenase (LDH) inhibitor oxamate and subsequently restored intracellular lactate levels by adding sodium lactate (Nala) (Fig. S1E). The results showed that oxamate treatment reduced H3K9la levels in DLD1 and CACO2 cells harboring mutant KRAS, while Nala supplementation rescued H3K9la levels in both oxamate-treated cells and KRAS knockdown SW480 and SW620 cells (Fig. S1F). To further validate this, we conducted experiments in KRAS mutant cell lines HCT116, DLD1, and LOVO. Lactate production was inhibited using (1) siRNAs targeting LDHA and LDHB or (2) the LDH inhibitor oxamate, while lactate levels were restored with Nala (Fig. 2G, H). Both LDH-specific siRNAs and oxamate significantly reduced H3K9la and lactylation levels, whereas Nala supplementation restored the decreased H3K9la and lactylation levels (Fig. 2I, J). Collectively, these results indicated that KRAS mutation increased lactate production to elevate H3K9la levels in CRC cells.

### Inhibition of H3K9la and lactylation suppressed proliferation and migration of CRC

**Fig. 3:**
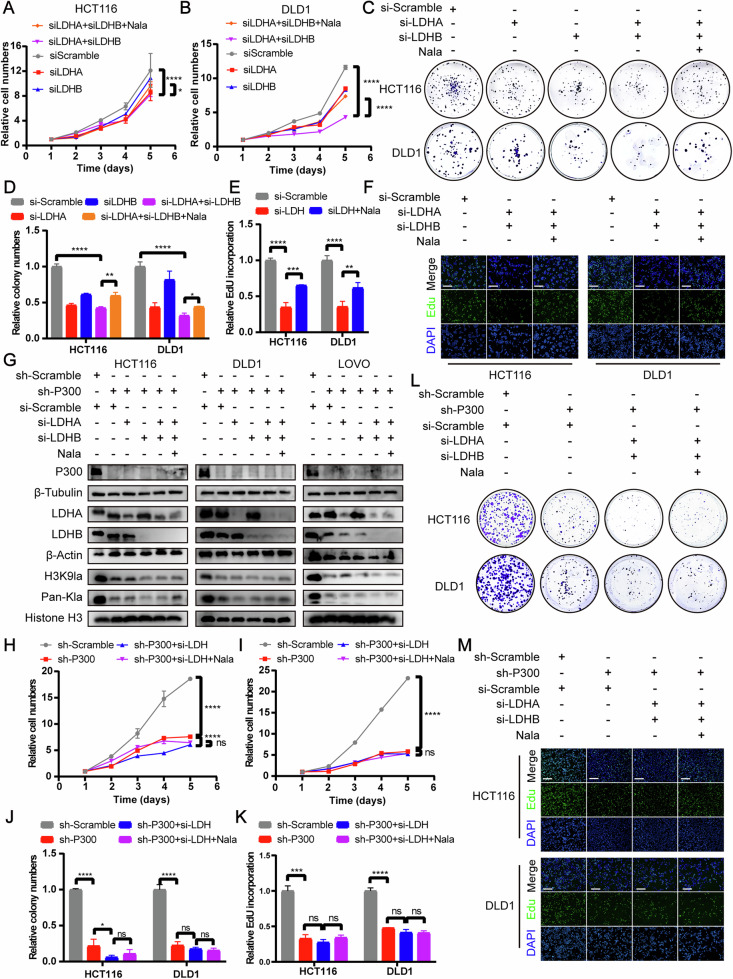
Inhibition of H3K9la and lactylation by silencing LDH suppressed proliferation of CRC. Proliferation of **A** HCT116 and **B** DLD1 cells after silencing LDHA, LDHB or both analyzed by CCK8 assay. *n*  =  4. **C**, **E** Tumor growth of HCT116 and DLD1 cells after silencing LDHA, LDHB or both evaluated by **C** colony formation assay and **E** statistical analysis. *n*  =  3. Proliferation of HCT116 and DLD1 cells after silencing both LDHA and LDHB evaluated by **D** Edu incorporation assay and **F** statistical analysis. *n*  =  3. **G** H3K9la and lactylation levels of P300 knock-down HCT116, DLD1 and LOVO cells after silencing both LDHA and LDHB. Proliferation of P300 knock-down **H** HCT116 and **I** DLD1 cells after silencing both LDHA and LDHB analyzed by CCK8 assay. *n*  =  4. Tumor growth of P300 knock-down HCT116 and DLD1 cells after silencing both LDHA and LDHB evaluated by **J** colony formation assay and **L** statistical analysis. *n*  =  3. Proliferation of P300 knock-down HCT116 and DLD1 cells after silencing both LDHA and LDHB evaluated by **K** Edu incorporation assay and **M** statistical analysis. *n*  =  3. Values are presented as mean ± SD. *p < 0.05, **p < 0.01, ***p < 0.001, ****p < 0.0001, ns *p* > 0.05, determined by one-way ANOVA (**A**, **B**, **D**, **E**, **H**–**K**).

To investigate the biological functions of H3K9la in KRAS mutant CRC, we utilized KRAS mutant cell lines HCT116, DLD1, and LOVO. H3K9la and lactylation were inhibited using oxamate or siRNAs targeting LDHA and LDHB as previously described. Cell proliferation assay, colony formation assay and Edu incorporation assay indicated that inhibition of H3K9la and lactylation suppressed proliferation and colony formation of CRC cells (Figs. 3A–F and S2A–D). Transwell assay and wound healing assay showed that inhibition of H3K9la and lactylation suppressed migration of CRC cells (Figs. S2E–H and S3A, B, E, F). Notably, restoration of H3K9la and lactylation with Nala partially rescued the proliferation and migration of CRC cells. To determine whether these biological effects were mediated by H3K9la and lactylation rather than lactate itself, we knocked down the lactylation writer P300 using shRNA to block the lactylation process independently of lactate levels. P300 knockdown significantly reduced H3K9la and lactylation levels, and these reductions were minimally affected by manipulating lactate levels with LDH siRNA and Nala (Fig. 3G). Similarly, after P300 knockdown, the proliferation (Fig. 3H–M) and migration (Fig. S3C, D, G, H) of CRC cells were inhibited and barely influenced by lactate concentration. These findings indicated that blocking H3K9la and lactylation by P300 knock-down partially diminished the biological functions of lactate on proliferation and migration of CRC cells. In vivo, we established xenografts in nude mice by subcutaneously transplanting LDHA- and LDHB-silenced HCT116 cells or control cells. As expected, the tumor volume (Fig. S4A, B), weight (Fig. S4C) and Ki-67 level in the reduced H3K9la group was significantly lower than that in control group (Fig. S4D–F). Taken together, these results revealed that inhibition of H3K9la and lactylation suppressed proliferation and migration of CRC cells.

### Increased H3K9la promoted GRAMD1A transcription in KRAS mutant CRC

**Fig. 4:**
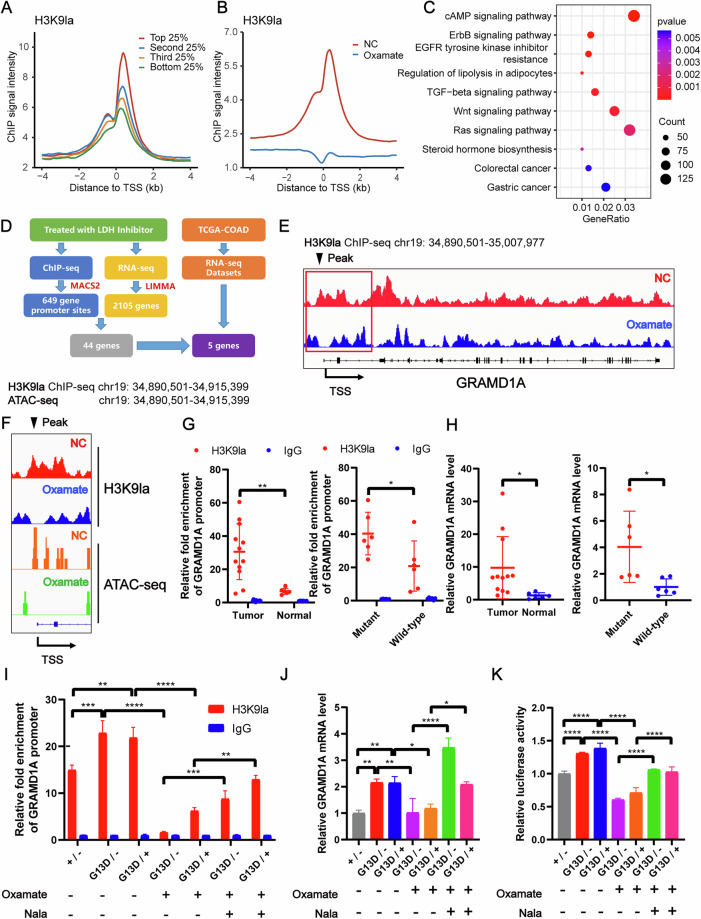
Increased H3K9la promoted GRAMD1A transcription in KRAS mutant CRC. **A** H3K9la correlated with steady-state mRNA levels. The average ChIP signal intensity (read count per million mapped reads) for indicated antibodies is shown for genes with different expression levels (the top 25%, the second 25%, the third 25%, and the bottom 25% of RNA-seq counts). **B** Distribution and level of H3K9la sites relative to translation start site (TSS) with or without LDH inhibitor oxamate treatment. **C** Kyoto Encyclopedia of Genes and Genomes (KEGG) analysis of differential H3K9la peaks with or without oxamate treatment. **D** Bioinformatics analysis filtered GRAMD1A as a downstream target of H3K9la. **E** IGV tracks for GRAMD1A from H3K9la ChIP-seq analysis. **F** IGV tracks for GRAMD1A promoter region from H3K9la ChIP-seq and ATAC-seq analysis. **G** H3K9la levels at GRAMD1A promoter region by ChIP-qPCR assay and **H** GRAMD1A mRNA levels by qPCR of KRAS mutant and wild-type CRC tissues and normal colon tissues. n  =  3. **I** H3K9la levels at GRAMD1A promoter region by ChIP-qPCR assay, **J** GRAMD1A mRNA levels by qPCR and **K** luciferase activity by dual luciferase reporter assay of isogenic DLD1 cells harboring G13D mutant, wild-type or both alleles of KRAS treated with 10 mM oxamate and 10 mM Nala. *n*  =  3. Values are presented as mean ± SD. *p < 0.05, **p < 0.01, ***p < 0.001, ****p < 0.0001, determined by two-tailed Student’s t-test (**G**, **H**) and one-way ANOVA (**I**–**K**).

To investigate the regulatory role of H3K9la in gene transcription, we conducted chromatin immunoprecipitation followed by sequencing (ChIP-seq) with antibodies against H3K9la, histone H3 lysine 9 acetylation (H3K9ac), and histone H3 lysine 9 trimethylation (H3K9me3), along with paired RNA sequencing (RNA-seq), in HCT116 cells treated with the LDH inhibitor oxamate and untreated controls. Combined analysis of ChIP-seq and RNA-seq results revealed that H3K9la and H3K9ac were enriched in promoter regions (±2 kb around transcriptional start sites), and genes with higher mRNA levels exhibited higher H3K9la and H3K9ac levels (Figs. 4A and S5A), indicating that H3K9la, like H3K9ac, was associated with active mRNA transcription, in contrast to H3K9me3 (Fig. S5B). Oxamate treatment significantly reduced H3K9la levels in promoter regions (Fig. 4B). Kyoto Encyclopedia of Genes and Genomes (KEGG) analysis of ChIP-seq data indicated that H3K9la-specific genes were enriched in cancer-related and lipid metabolism pathways (Fig. 4C). Gene ontology (GO) enrichment analysis of RNA-seq data revealed that downregulated genes after oxamate treatment were also enriched in lipid metabolism and cell growth pathway (Fig. S5C). By integrating our ChIP-seq and RNA-seq data with transcriptome of normal and CRC tissues in TCGA database, we identified five differentially expressed genes (GRAMD1A, PTK7, SPARC, SPHK1 and TMEM241) potentially regulated by H3K9la (Fig. 4D, Fig. S5D). Among them, GRAM domain-containing protein 1A (GRAMD1A), a non-vesicular cholesterol transporter protein, exhibited significant H3K9la enrichment in its promoter region (Fig. 4E), reduced mRNA transcription after LDH inhibitor treatment (Fig. S5D), and higher expression in CRC tissues compared to normal colon tissues. ChIP-qPCR confirmed H3K9la enrichment at the GRAMD1A promoter, which was decreased by oxamate and partially rescued by Nala (Fig. S5E, F). Similarly, mRNA level of GRAMD1A was decreased by oxamate treatment and increased by Nala addition (Fig. S5G, H). These findings suggested that H3K9la promoted GRAMD1A transcription.

**Fig. 5:**
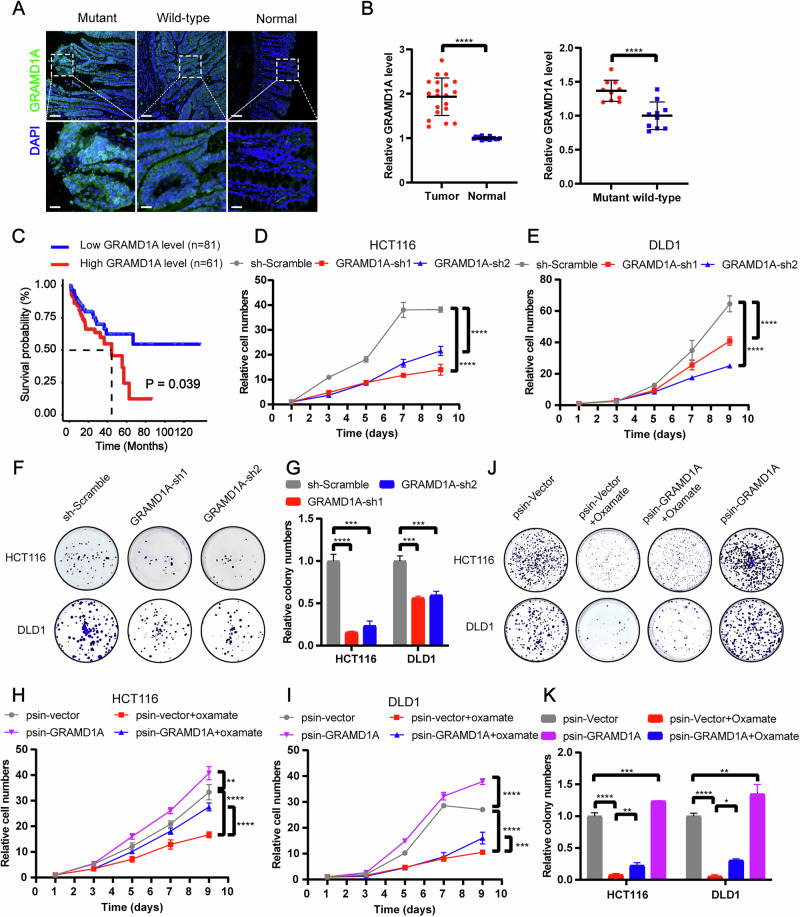
GRAMD1A promoted proliferation of CRC. **A** Immunofluorescence staining of GRAMD1A expression in KRAS mutant and wild-type CRC tissues and normal colon tissues. Scale bar: up panel, 100 μm; down panel 25 μm. **B** Statistical immunofluorescence results of GRAMD1A expression in KRAS mutant and wild-type CRC tissues and normal colon tissues. **C** Kaplan–Meier survival curves contrasting difference between CRC patients in advanced stages with low and high GRAMD1A expression from TCGA datasets. Proliferation of **D** HCT116 and **E** DLD1 cells after knocking down GRAMD1A analyzed by CCK8 assay. *n*  =  4. **F**, **G** Tumor growth of HCT116 and DLD1 cells after knocking down GRAMD1A evaluated by **F** colony formation assay and **G** statistical analysis. *n*  =  3. Proliferation of **H** HCT116 and **I** DLD1 cells after GRAMD1A overexpression with or without oxamate treatment analyzed by CCK8 assay. *n*  =  4. Tumor growth of HCT116 and DLD1 cells after GRAMD1A overexpression with or without oxamate treatment analyzed by **J** colony formation assay and **K** statistical analysis. *n*  =  3. Values are presented as mean ± SD. *p < 0.05, **p < 0.01, ***p < 0.001, ****p < 0.0001, determined by two-tailed Welch’s t-test (left) or Student’s t-test (right) (**B**), Log-rank test (**C**) and one-way ANOVA (**D**, **E**, **G**, **H**, **I**, **K**).

To further elucidate the functional role of H3K9la in GRAMD1A transcription, we initially hypothesized that H3K9la might influence transcription by competing with H3K9ac or H3K9me3, since these modifications occur at the same lysine site on histone H3 as H3K9la and are known regulators of gene transcription [16]. However, our ChIP-seq results showed that after oxamate treatment, the decreased H3K9la level (Fig. 4B, E) had no obvious influence on H3K9ac or H3K9me3 levels either at the GRAMD1A promoter region (Fig. S6A) or globally (Fig. S6B, C). These findings suggested that the transcriptional effects of H3K9la were likely independent of H3K9ac and H3K9me3. Given that DNA is organized into nucleosomes to form chromatin, and the compact structure of chromatin restricts transcription factor access to DNA, therefore chromatin accessibility was a prerequisite to the execution of transcription [17]. Various epigenetic modifications, including acylation and methylation, have been shown to regulate transcription by modulating chromatin accessibility [18, 19]. To examine whether H3K9la influences chromatin accessibility, we performed assay for transposase-accessible chromatin with high throughput sequencing (ATAC-seq) on HCT116 cells treated with oxamate. The results showed that oxamate treatment reduced chromatin accessibility both at GRAMD1A promoter region (Fig. 5F) and globally (Fig. S6D), correlating with decreased H3K9la levels. These data indicated that H3K9la positively regulated GRAMD1A transcription by enhancing chromatin accessibility rather than through competition with H3K9ac or H3K9me3.

Given our previous findings that KRAS mutations promoted lactate production to elevate H3K9la levels, we hypothesized that KRAS mutations in CRC promote GRAMD1A transcription via increased H3K9la levels. ChIP-qPCR and paired qPCR analyses of KRAS mutant, KRAS wild-type CRC, and normal colon tissues revealed both significantly higher H3K9la levels at the GRAMD1A promoter (Fig. 4G) and elevated GRAMD1A mRNA levels (Fig. 4H) in KRAS-mutant CRC tissues compared to KRAS wild-type CRC tissues, and in CRC tissues compared to normal colon tissues. To confirm that the upregulation of GRAMD1A in KRAS-mutant CRC is mediated by H3K9la, we manipulated H3K9la levels at GRAMD1A promoter (Fig. 4I) and assessed GRAMD1A transcription using qPCR (Fig. 4J) and dual luciferase reporter assays (Fig. 4K) in isogenic DLD1 cell lines. The results indicated that KRAS mutation increased H3K9la level at GRAMD1A promoter, leading to upregulation of GRAMD1A mRNA and transcriptional activity, while inhibition of H3K9la decreased GRAMD1A transcription in KRAS-mutant cells, which partially rescued by restoring H3K9la. Additionally, the KRAS mutation had no significant impact on the binding of P300, HDAC1, HDAC2, and HDAC3 to the promoter region of GRAMD1A, where KRAS mutation was observed to increase H3K9la levels (Fig. S6E–H). Collectively, these findings suggested that KRAS mutations promoted GRAMD1A transcription by increasing H3K9la.

### GRAMD1A promoted tumor growth and metastasis in CRC

Given our previous findings that GRAMD1A can be upregulated by mutant KRAS-mediated H3K9la, we next investigated its role in CRC. Immunofluorescence staining (Fig. 5A, B) and transcriptome data in TCGA database (Fig. S7A, B) showed that GRAMD1A expression was significantly higher in KRAS mutant CRC compared to KRAS wild-type CRC, and in CRC tissues compared to normal colon tissue. Kaplan-Meier and Cox analysis of TCGA data indicated higher expression of GRAMD1A was positively correlated with poor prognosis in advanced clinical stages and could serve as an independent predictor of survival (Figs. 5C and S7C). To elucidate the functional role of GRAMD1A, we established GRAMD1A knockdown and overexpression cell lines from HCT116, DLD1, and LOVO cells (Fig. S8A–D). GRAMD1A knockdown suppressed CRC cell proliferation (Figs. 5D–G and S8E, F) and migration (Fig. S9A–D), whereas GRAMD1A overexpression promoted proliferation (Figs. 5H–K and S8G, H) and migration (Fig. S9E–H), partially counteracting the anticancer effects of inhibiting H3K9la and lactylation by oxamate. These results suggest that H3K9la promotes CRC progression partially through GRAMD1A.

**Fig. 6:**
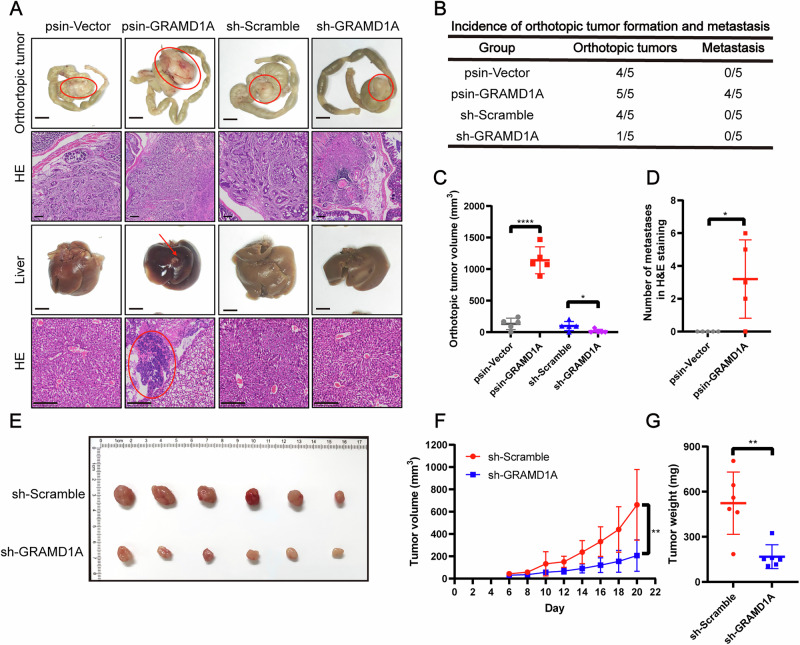
GRAMD1A promoted growth and metastasis of CRC in vivo. **A** Representative images of gross inspection and H&E staining of the CRC orthotopic tumors or liver metastatic tumors (*n* = 5 per group). Orthotopic tumors scale bar: 0.5 cm; H&E scale bars: 250 μm. **B** Orthotopic xenograft CRC tumor formation and liver metastasis analysis. **C** Statistical analysis of orthotopic tumor volumes. **D** Statistical analysis of number of metastases in H&E staining of liver. **E** Representative images of gross inspection of subcutaneous xenografts of CRC. Statistical analysis of subcutaneous tumor **F** volumes and **G** weights. Values are presented as mean ± SD. *p < 0.05, **p < 0.01, ****p < 0.0001, determined by Mann–Whitney test (**C**, **D**, **F**, **G**).

To further validate the role of GRAMD1A in vivo, we established orthotopic xenograft models by injecting LOVO cells knocked-down or overexpressed GRAMD1A to observe the tumor growth and liver metastasis. Our results showed that GRAMD1A overexpressing group showed 100% (5/5) tumor formation and 80% (4/5) liver metastasis, compared to 80% (4/5) tumor formation and 0% (0/5) liver metastasis in vector group. In contrast, GRAMD1A knockdown group showed 20% (1/5) tumor formation and 0% (0/5) liver metastasis, compared to 80% (4/5) tumor formation and 0% (0/5) liver metastasis in scramble group (Fig. 6A, B). Additionally, GRAMD1A overexpressing group showed significantly higher tumor volumes (Fig. 6C) and liver metastasis numbers in H&E staining (Fig. 6D) than the vector group, while GRAMD1A knockdown group had significantly lower tumor volumes compared to scramble group (Fig. 6C). Moreover, our subcutaneous xenograft tumor models using HCT116 cells also revealed that GRAMD1A knockdown reduced tumor volumes and weight in vivo (Fig. 6E–G). In addition, subcutaneous xenograft tumor models using DLD1 cells harboring mutant (G13D/−), wild-type (+/–) or both KRAS alleles (G13D/+) further indicated that GRAMD1A knock-down showed more pronounced effect on tumor volume and weight in KRAS mutant tumors (Fig. S10A–D). Taken together, these data indicated that GRAMD1A promoted proliferation and metastasis of CRC cells.

### GRAMD1A promoted CRC progression through facilitating cholesterol metabolism

**Fig. 7:**
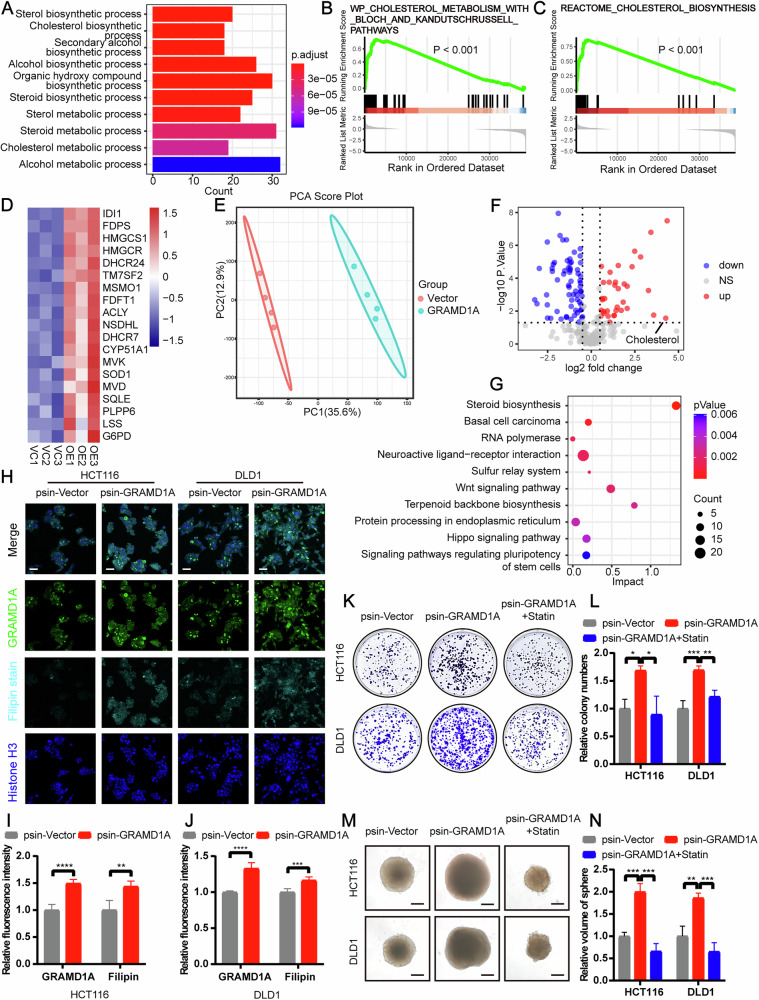
GRAMD1A promoted CRC proliferation through facilitating cholesterol metabolism. **A** Gene ontology (GO) enrichment analysis of up-regulated genes detected by RNA-seq after GRAMD1A overexpression in HCT116 cells. **B**, **C** Gene set enrichment analysis (GSEA) of cholesterol metabolism and biosynthesis pathways after GRAMD1A overexpression in HCT116 cells. **D** Heatmap of mRNA level determined by RNA-seq of enzymes involved in de novo cholesterol biosynthesis in HCT116 cells overexpressing GRAMD1A or vector. **E** Principal component analysis (PCA) score plot and **F** volcano plot of metabolites determined by metabolomics in HCT116 cells overexpressing GRAMD1A or vector. **G** Combined KEGG analysis of RNA-seq and metabolomics of HCT116 cells overexpressing GRAMD1A or vector. **H** Filipin staining of cholesterol levels and immunofluorescence staining of GRAMD1A expression in HCT116 and DLD1 cells overexpressing GRAMD1A or vector. Scale bar: 50 μm. Statistical immunofluorescence results of cholesterol levels and GRAMD1A expression in **I** HCT116 and **J** DLD1 cells. *n*  =  5. Tumor growth of HCT116 and DLD1 cells after GRAMD1A overexpression and 5 μM Simvastatin treatment evaluated by **K** colony formation assay and **L** statistical analysis. *n*  =  3. Tumor growth of HCT116 and DLD1 cells after GRAMD1A overexpression and 5 μM Simvastatin treatment evaluated by **M** sphere formation assay and **N** statistical analysis. Scale bar: 500 μm. *n*  =  3. Values are presented as mean ± SD. *p < 0.05, **p < 0.01, ***p < 0.001, ****p < 0.0001, determined by two-tailed Student’s t-test (**I**, **J**) and one-way ANOVA (**L**, **N**).

To understand the mechanism of GRAMD1A in CRC progression, we conducted RNA-seq on GRAMD1A-overexpressing and vector control HCT116 cells. GO analysis showed that genes upregulated in GRAMD1A-overexpressing cells were enriched in steroid and cholesterol metabolism pathway (Fig. 7A). In addition, gene set enrichment analysis (GSEA) indicated that GRAMD1A overexpression significantly enhanced cholesterol metabolism and biosynthesis pathways (Fig. 7B, C), with upregulated mRNA levels of enzymes involved in de novo cholesterol biosynthesis (Fig. 7D). To further validate these findings, we conducted metabolomic analysis on GRAMD1A-overexpressing and vector control HCT116 cells using liquid chromatograph-mass spectrometer (LC–MS). Principal component analysis (PCA) showed significant differences in metabolomic characteristics between the GRAMD1A-overexpressing and vector control groups (Fig. 7E), with a marked increase in cholesterol levels in GRAMD1A-overexpressing cells compared to vector control cells (Figs. 7F and S11A). Combined KEGG analysis of metabolomic and RNA-seq results further validated that steroid biosynthesis and cancer related pathways were enriched in GRAMD1A-overexpressing cells (Fig. 7G). Moreover, cholesterol staining by Filipin complex showed higher cholesterol levels in GRAMD1A overexpressing cells (Fig. 7H–J). As SREBP2 is the key transcription factor regulating the expression of genes involved in cholesterol metabolism [20], we further investigated whether GRAMD1A affects SREBP2. Western blot analysis showed that GRAMD1A overexpression increased the protein level of the nuclear form (nSREBP2) but not the precursor form of SREBP2 (pSREBP2) (Fig. S11B). In addition, given that HMG-CoA reductase (HMGCR) is the rate-limiting enzyme in the cholesterol biosynthesis (mevalonate) pathway and its transcription is mainly regulated by SREBP2 [21], we cloned the promoter region of HMGCR into a dual luciferase reporter plasmid. The dual-luciferase reporter assay showed that GRAMD1A overexpression increased the relative luciferase activity driven by the HMGCR promoter, suggesting that GRAMD1A overexpression enhanced the transcriptional activity of SREBP2 (Fig. S11C, D). These findings indicated that GRAMD1A facilitated cholesterol metabolism in CRC.

To determine whether inhibition of cholesterol metabolism could compromise the effect of GRAMD1A overexpression, we utilized the cholesterol biosynthesis inhibitor Simvastatin. Our results showed that Simvastatin partially attenuated the promotion of cellular proliferation (Figs. 7K–N and S11E, F) and migration (Fig. S11G–J) induced by GRAMD1A overexpression in CRC cells. Collectively, these results indicated that GRAMD1A promoted proliferation and migration in CRC by facilitating cholesterol metabolism.

### Inhibition of H3K9la or GRAMD1A suppressed the growth of CRC patient-derived xenografts

**Fig. 8:**
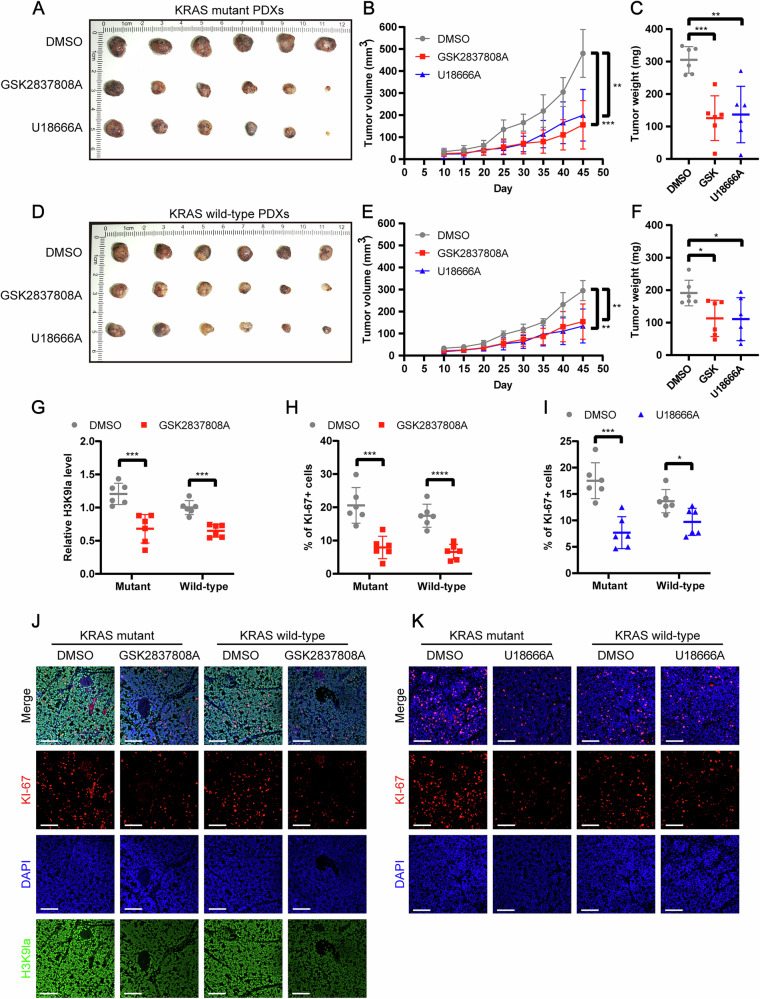
Inhibition of lactylation or GRAMD1A suppressed the growth of CRC patient-derived xenografts (PDX). **A** Image of KRAS mutant PDX treated with GSK2837808A or U18666A. Statistical analysis of tumor **B** volume and **C** weight. **C** Image of KRAS wild-type PDX treated with GSK2837808A or U18666A. Statistical analysis of tumor **D** volume and **E** weight. **J** Immunofluorescence staining of H3K9la levels and Ki-67 expression in KRAS mutant and wild-type PDX treated with GSK2837808A. Scale bar: 100 μm. Statistical immunofluorescence results of **G** H3K9la levels and **H** Ki-67 expression. **K** Immunofluorescence staining of Ki-67 expression in KRAS mutant and wild-type PDX treated with U18666A. Scale bar: 100 μm. Statistical immunofluorescence results of **I** Ki-67 expression. Values are presented as mean ± SD. *p < 0.05, **p < 0.01, ***p < 0.001, ****p < 0.0001, determined by one-way ANOVA (**B**, **C**, **E**, **F**) and two-tailed Student’s t-test (**G**–**I**).

To evaluate the potential therapeutic value of targeting H3K9la and GRAMD1A, we constructed KRAS mutant and wild-type CRC PDX mouse models. The mice were treated with solvent control (dimethyl sulfoxide, DMSO), LDH inhibitor (GSK2837808A), or GRAMD1A inhibitor (U18666A). Our results showed that both LDH inhibitor and GRAMD1A inhibitor treatments significantly reduced tumor volumes and weight in CRC PDX models (Fig. 8A–F). Immunofluorescence analysis of the PDX samples revealed that the LDH inhibitor significantly decreased H3K9la levels (Fig. 8G, J). Furthermore, both inhibitors suppressed cellular proliferation, as evidenced by a decrease in the proportion of Ki-67-positive cells (Fig. 8H–K). Notably, a more pronounced therapeutic effect was observed in KRAS-mutant PDX compared to KRAS wild-type PDX. Collectively, these results highlighted the crucial roles of H3K9la and GRAMD1A in CRC progression and underscored their potential therapeutic value, particularly in KRAS-mutant CRC.

## Discussion

The mechanisms by which KRAS regulated gene expression and various aspects of tumor biology through signaling transduction pathways were well-documented. The key findings of our current study presented important perspectives on the role of KRAS in regulating H3K9la, a novel epigenetic modification, to promote malignant progression in CRC. We discovered that CRC cells harboring mutant KRAS exhibited elevated levels of lactylation and H3K9la. Inhibition of aberrant H3K9la efficiently suppressed malignant progression. Mechanistically, mutationally activated KRAS drove the production of lactate in tumor cells, which then functioned as a substrate to increase H3K9la level. Elevated H3K9la promoted the expression of GRAMD1A, a cholesterol transporter, by increasing chromatin accessibility at its promoter region. GRAMD1A facilitated cholesterol biosynthesis, thereby promoting the growth and metastasis of CRC. Identification of these interactions between KRAS and H3K9la provided a better understanding of the mechanisms by which KRAS affects CRC progression, and might provide novel insights on potential therapeutic strategies for treating KRAS mutant tumors.

KRAS is one of the RAS superfamilies belonging to the group of small-guanosine triphosphate (GTP) binding proteins. Oncogenic mutations in KRAS drive common metabolic changes across colorectal, lung, and pancreatic cancers, which facilitate tumor survival, growth and immune evasion. Notably, cancer cells harboring KRAS mutation highly expressed glucose transporters and glycolytic enzymes, leading to rapid anerobic glycolysis to produce lactate [6]. The association between KRAS and lactate production has been well established [22]. Consistent with previous studies, our findings for the first time showed that KRAS increased lactylation and H3K9la by enhancing lactate production. Importantly, this KRAS-mediated increase in histone lactylation was not achieved through alterations in levels of P300, HDAC1, HDAC2, or HDAC3, the key enzymes responsible for writing or erasing histone lactylation. Furthermore, elevation of lactylation and H3K9la levels is unlikely to correlate with KRAS mutation type. Inhibition of lactylation and H3K9la efficiently suppressed KRAS mutant CRC proliferation and migration.

Post-translational modifications of histones serve as crucial regulators of gene expression, modulating a variety of biological processes by creating docking sites for recruiting chromatin modulators. Abnormalities in various histone modifications are commonly associated with several human diseases, including cancer, highlighting the importance of histone-based gene regulation [23]. A novel form of histone modification, lysine lactylation, has recently emerged as a key player in gene regulation. Researchers have identified 28 distinct potential lactylation sites on core histone proteins, with notable examples including H3K9la, H3K14la, and H3K18la. Recent studies uncovered that Increased H3K18la in promoter regions triggered the expression of genes implicated in tumorigenesis, cancer progression and macrophage polarization. But the role of other lactylation sites remained to be fully explored. In line with previous studies, we for the first time revealed that H3K9la in promoter regions could induce the transcription of genes related to metabolism and tumor progression by increasing chromatin accessibility. Moreover, it has been well-established that mutations in KRAS, a Ras-like GTPase, constitutively activate receptor tyrosine kinase pathways, promoting the expression of genes involved in various aspects of tumor biology. Our findings revealed that KRAS also increased H3K9la, thereby facilitating the expression of cancer-related genes and driving malignant progression. This uncovered a potential role for KRAS in regulating cancer epigenetics.

Cholesterol homeostasis plays a crucial role in regulating both cellular and systemic metabolism, and its dysregulation has been increasingly linked to cancer development. Cholesterol tends to accumulate in cancer cells due to abnormal expression of genes involved in cholesterol metabolism, which in turn promotes proliferation, invasion, and immune evasion [24]. In CRC, enhanced cholesterol metabolism by upregulation of squalene epoxidase (SQLE), a rate-limiting enzyme in cholesterol biosynthesis, promoted CRC cell proliferation by inducing cell cycle progression and suppressing apoptosis [25]. Additionally, cholesterol accumulation accelerated CRC progression and metastasis by inducing the epithelial-mesenchymal transition (EMT) [26]. GRAMD1A, a member of the Aster/GRAMD1 family of non-vesicular cholesterol transporters, plays a pivotal role in maintaining cholesterol homeostasis. It senses plasma membrane cholesterol levels and facilitates the transport of accessible cholesterol to the endoplasmic reticulum, a key site for controlling cholesterol biosynthesis and uptake. By facilitating this movement of cholesterol, GRAMD1A helps to modulate the cholesterol metabolism in cells [27]. In our study, we revealed that KRAS-mediated H3K9la elevation upregulated the expression of GRAMD1A, thereby promoting CRC growth and metastasis by enhancing cholesterol biosynthesis. Moreover, inhibiting cholesterol biosynthesis compromised the effect of GRAMD1A on CRC progression. Interestingly, the intermediate products of the mevalonate pathway in de novo cholesterol biosynthesis, farnesyl pyrophosphate (FPP) and geranylgeranyl pyrophosphate (GGPP), are essential for the prenylation of RAS proteins, a post-translational modification that enables their plasma membrane localization and functions [28]. This suggested a potential positive feedback loop where KRAS mutations upregulate GRAMD1A via H3K9la, promoting cholesterol biosynthesis, which in turn enhances KRAS prenylation, further driving KRAS signaling and H3K9la.

However, several limitations of this study should be acknowledged. First, it has not been detected in this study that whether KRAS mutations influence the enzymatic activity of key lactylation-related enzymes, such as P300 and HDAC1-3. KRAS mutations profoundly alter cellular protein phosphorylation through the constitutive activation of signaling pathways [4], and the enzymatic activities of P300, HDAC-3 are known to be regulated by phosphorylation [29, 30], further studies are warranted to determine whether KRAS mutations also modulate the activity of these enzymes. Second, the current methods used to manipulate H3K9la levels lack specificity. Inhibition of lactate dehydrogenases affects both cellular metabolism and global protein lactylation [7, 31], while P300 knockdown impacts not only histone lactylation but also the acetylation and lactylation of other proteins [7, 29]. Future studies should identify the specific motif responsible for H3K9la deposition and selectively inhibit it via point mutation. Third, the GRAMD1A inhibitor U18666A lacks specificity and also targets Niemann-Pick C1 (NPC1), a key protein involved in vesicular trafficking [32]. Future studies should employ more selective agents, such as the U18666A analog AI-3d [32], other specific inhibitors, to better evaluate the therapeutic potential in PDX models. Finally, although we have shown that GRAMD1A overexpression increases nuclear SREBP2 (nSREBP2) levels, the precise mechanism by which GRAMD1A promotes cholesterol metabolism remains to be elucidated. Whether this occurs through direct modulation of SREBP2 processing or via alternative pathways requires further investigation.

In conclusion, our study revealed that KRAS mutation elevated lactylation and H3K9la levels, by promoting lactate production. This upregulation in H3K9la increased chromatin accessibility at the GRAMD1A promoter, thereby facilitating its transcription. Subsequently, GRAMD1A drove CRC growth and metastasis by promoting cholesterol biosynthesis. These findings suggested that targeting H3K9la and GRAMD1A could be a promising therapeutic strategy for KRAS-mutant CRC, which demonstrated remarkable efficacy in CRC PDX models.

## Supplementary information

Supplementary Methods and Figures

Supplement Material-uncropped western blots

## Data Availability

The ChIP-seq, RNA-seq, ATAC-seq and metabolomics datasets used during the current study are available from the corresponding author on reasonable request. The TCGA datasets analyzed during the current study are open to the public at the cBioPortal online database (http://www.cbioportal.org).
