# Supplementary material for: KRAS mutation increases histone H3 lysine 9 lactylation (H3K9la) to promote colorectal cancer progression by facilitating cholesterol transporter GRAMD1A expression
Source: Cell Death Differ. 2025 Jul 24;32(12):2225–38. doi: 10.1038/s41418-025-01533-4 (PMC12669710; doi:10.1038/s41418-025-01533-4)
Supplement: Supplementary file 1 — Supplementary Methods and Figures [file 41418_2025_1533_MOESM1_ESM.docx]

# Materials and methods

## Immunofluorescence and Filipin staining

Formalin-fixed, paraffin-embedded human CRC and normal colon tissues, along with mouse xenograft and PDX tissue sections, were deparaffinized, rehydrated, fixed, and blocked with 5% bovine serum albumin (BSA). CRC cells cultured on confocal dishes were fixed with 4% paraformaldehyde (PFA), then blocked and permeabilized using 5% BSA containing 0.3% Triton X-100. The tissues and cells were incubated overnight at 4°C with primary antibodies: anti-Pan-Kla (PTM, #PTM-1401), anti-H3K9la (PTM, #PTM-1419RM), anti-P300 (Abcam, #ab275378), anti-HDAC1 (Abcam, #ab109411), anti-HDAC2 (Abcam, #ab124974), anti-HDAC3 (Abcam, #ab32369), anti-GRAMD1A (Novus, #NBP1-93729), and anti-Ki-67 (Abcam, #ab15580). The slides were then incubated with appropriate secondary antibodies for 2 hours, followed by nuclear staining with DAPI (Sigma-Aldrich) for 30 minutes. For Filipin staining, after fixation, blocking, and permeabilization, cancer cells were incubated with 0.1 mg/ml Filipin (MCE, #HY-N6716) in PBS containing 10% BSA for 1 hour. Digital images were captured using confocal microscopes (ZEISS LSM 880, Munich, Germany and Leica SP8, Durham, USA). Relative Pan-Kla, H3K9la, and cholesterol levels, as well as P300, HDAC1, HDAC2, HDAC3, GRAMD1A, and Ki-67 expression, were quantified by comparing the fluorescence mean intensity of the target antibodies using ImageJ software.

## Western blotting

Cell and tissue samples were lysed using radio-immunoprecipitation assay (RIPA) buffer supplemented with a protease and phosphatase inhibitor cocktail (Promega). Proteins were separated by SDS-PAGE and transferred to polyvinylidene fluoride (PVDF) membranes using the Trans-Blot System (Bio-Rad, CA, USA). The membranes were blocked with 5% BSA and then incubated with primary antibodies: anti-Pan-Kla (PTM, #PTM-1401), anti-H3K9la (PTM, #PTM-1419RM), anti-H3K14la (PTM, #PTM-1414RM), anti-H3K18la (PTM, #PTM-1406), anti-P300 (Abcam, #ab275378), anti-HDAC1 (Abcam, #ab109411), anti-HDAC2 (Abcam, #ab124974), anti-HDAC3 (Abcam, #ab32369), anti-GRAMD1A (Novus, #NBP1-93729), anti-Histone H3 (Affinity, #AF0863), anti-β-Actin (Affinity, #AF7018), anti-ERK1/2 (Affinity, #AF0155), anti-Phospho-ERK1/2 (Affinity, #AF1015), anti-pSREBP2 (Affinity, #DF7601), anti-nSREBP2 (Invitrogen, # PA5-88943), anti-β-Tubulin (Affinity, #AF7011), anti-LDHA (Proteintech, #19987-1-AP), and anti-LDHB (Proteintech, #19988-1-AP). Finally, the membranes were incubated with specific secondary antibodies and visualized using ECL Blotting Detection Reagents. All of full and uncropped western blots were uploaded as Supplement Material-uncropped western blots.

## Immunoprecipitation assay and mass spectrometry

For the immunoprecipitation assay, HCT116 cells were lysed on ice in Pierce IP lysis buffer (Beyotime, #P0013J) containing a protease inhibitor cocktail (Roche, #4693116001). Cell lysates were centrifuged at 13,000g for 20 minutes at 4°C to obtain the protein-containing supernatant. Protein A/G Magnetic Beads (Thermo Fisher, #88802) were prewashed twice with washing buffer and crosslinked with anti-Pan-Kla (PTM, #PTM-1401) for 4 hours at 4°C. The protein sample was then incubated with the antibody-crosslinked beads overnight at 4°C. The beads were washed four times with washing buffer, followed by incubation with 2x SDS loading buffer at 100°C for 10 minutes. The resultant samples were resolved by SDS-PAGE and stained with Coomassie Brilliant Blue. The target band was excised and processed for liquid chromatography-tandem mass spectrometry (LC-MS/MS) at FitGene Biotechnology (Guangzhou, China).

## Cell culture and treatment

Human CRC cell lines (HCT116, SW480, SW620, DLD1, CACO2, LOVO, RKO, KRAS isogenic DLD1) and the HIEC-6 cell line were obtained from the American Type Culture Collection (ATCC). Cells were cultured in DMEM or RPMI 1640 medium (Gibco, NY, USA) supplemented with 10% fetal bovine serum and 1% penicillin-streptomycin (Gibco, CA, USA) at 37 °C in a 5% CO2-humidified incubator. For oxamate treatment, 5 mM, 10 mM, and 20 mM oxamate (APExBIO, #C3893) were added to the culture medium. For lactate restoration, 5 mM or 10 mM sodium lactate (Sigma, #L4263) were used. For statin treatment, 5 µM or 10 µM Simvastatin (MCE, #HY-17502) was added to the culture medium.

## Lactate production assay

48 hours after indicated treatment, five million cells were extracted and lysed to measure intracellular lactate concentration using lactate assay kits (Abbkine, #KTB1100). All procedures were performed according to the manufacturer’s recommendations and cell counting was performed using a cell counter.

## Cell proliferation assays

Around 4000 cells were seeded into 96-well plates (Nest) with 200 μL of culture medium. Cell proliferation was analyzed by CCK-8 assays (APExBIO, #K1018) following the manufacturer’s instructions. The absorbance was detected at a wavelength of 450 nm, and growth curves were generated to determine the growth rates.

## Colony formation assay

After the indicated treatment, 1000 cells were seeded into 6-well plates and cultured at 5% CO2 at 37° C for 2 weeks. Then the cells were fixed with methanol and stained with 1% crystal violet for 15 min at room temperature. The colony numbers were calculated using QuPath-0.3.0 software.

## Edu incorporation assay

The Edu incorporation assay was performed using a Cell-Light EdU DNA Cell Proliferation Kit (Beyotime, #C0071L) following the manufacturer’s manual. The EdU-positive cells were imaged by inverted fluorescence microscopy (Olympus) and counted by Image J software.

## Transwell assay

Transwell assays were performed with 24-well plates (Corning) with 8-μm pore size chamber inserts (Falcon, #353097). In general, 1×10^5^ cells resuspend with 200 μl serum-free DMEM were seeded in the upper chamber well and 500 μl of DMEM with 10% FBS was added into the lower chamber. After 12 or 48 hours, cells migrating through the membrane were fixed with 4% paraformaldehyde for 30 min, and then stained with 0.1% crystal violet for 30 min. The cells were imaged under an inverted microscope (Olympus) and quantified using QuPath-0.3.0 software.

## Wound healing assay

A total of 2×10^6^ cells were seeded in six-well plates and incubated until confluency was reached. A 100ul pipette tip was used to create a rectilinear scratch. After 12 or 24 hours, the wound closure was imaged by an inverted microscope (Olympus) and then analysis by Image J software.

## ChIP assay

ChIP assay was performed using ChIP assay kits (Merck Millipore, #17-10085) following manufacture’s manual. Generally, HCT116 cells were treated with or without 10 mM oxamate for 24h, then fixed with 1% formaldehyde and lysed with cell lysis buffer to obtain the cell nucleus. Tissues stored in liquid nitrogen are lysed with beads and cell lysis buffer in a tissue lyser to obtain the cell nucleus. Ten million cell nucleus was sonicated for 14 min (15 s on and 45 s off) in 4 °C with a 40% output control. Sonicated chromatin fragments (50 μl) were diluted 10-fold and 1% of them was extracted as “Input”. Then, protein A/G magnetic beads and immunoprecipitating antibodies including anti-P300 (Abcam, #ab275378), anti-HDAC1 (Abcam, #ab280198), anti-HDAC2 (Abcam, #ab124974), anti-HDAC3 (Abcam, #ab137704), anti-H3K9la (PTM, #PTM-1419RM), anti-H3K9ac (Abcam, #ab32129), anti-H3k9me3 (Affinity, #DF6938), and IgG control (PTM, #PTM-6968) was added to the chromatin fragments and incubated overnight at 4 °C. The DNA was released from the bound chromatin after cross-linking reversal and proteinase K treatment, precipitated, and diluted. Purified DNA fragments were constructed and added to ChIP-seq libraries, amplified, and sequenced on an HiSeq 2500 platform (Illumina). The ChIP-seq experiments were reproduced once. The primers used for the qPCR analysis in this study are listed in Table S1.

## RNA sequencing

Total RNA was extracted from cultured cell samples using TRIzol reagent (Invitrogen, Carlsbad, CA, USA). Bioanalyzer 2200 instrument (Agilent, Santa Clara, CA, USA) was used to analyze RNA purity. Subsequently, mRNA was isolated, fragmented and processed for cDNA library construction. Finally, the cDNA library was sequencing and the obtained reads were aligned to GRCH38.p14 NCBI by Bowtie2 software. Limma packages were utilizing to analyze the differentially expressed genes.

## ATAC-seq

For the ATAC-seq, HCT116 cells were treated with or without 10 mM oxamate for 24h. Then, fifty thousand cells were harvested and subjected to ATAC-seq assay kit (Vazyme, #TD711-01). All procedures were performed following the manufacturer’s manual and cell was counted using a cell counter. Purified DNA fragments were constructed and added to ATAC-seq libraries, amplified, and sequenced on an HiSeq 2500 platform (Illumina). The ATAC-seq experiments were reproduced once.

## RNA isolation and quantitative real-time PCR (RT-PCR)

Total RNA was extracted from liquid nitrogen-stored tissues and cultured cells using TRIzol reagent (Invitrogen, Carlsbad, CA, USA). ReverTra Ace qPCR RT Kit (Toyobo) was applied for reverse transcription according to the instructions. Quantitative RT-PCR was performed using SYBR Green PCR master mix (ES Science) and a RT-PCR system (Applied Biosystems 7500 Sequence Detection, Singapore). ACTB was used as the control for PCR product quantification and normalization. The qRT-PCR primers for each gene are provided in Table S2.

## RNA Interference

siRNAs targeting human scramble, LDHA and LDHB were purchased from IGE Biotechnology (Guangzhou, China). Targeting sequences for LDHA were a pool of the following four target sequences: sequence 1, GGAGAA-AGCCGUCUUAAUU; sequence 2, GGCAAAGACUAUAAUGUAA; sequence 3, UAAGGGUCUUU-ACGGAAUA; and sequence 4, AAAGUCUUCUGAUGUCAUA. Targeting sequences for LDHB were a pool of the following two target sequences: sequence 1, AGATTGTAGTGGTAACTGC; sequence 2, GCAGCUGACU-UUGUCUUCU. siRNAs were premixed with Lipofectamine® 3000 (Invitrogen), and then added into the corresponding cells.

## Plasmids construction, transfection, and lentivirus infection

The coding sequence (CDS) of KRAS and GRAMD1A were cloned into the lentiviral expression plasmid named pSin-EF2-Puro (modified from pSin-EF2-Sox2-Puro, Addgene, #16577). For constructing different KRAS expression plasmids, the CDS of KRAS were amplificated from cDNA of LS174T (KRAS G12D), SW620 (KRAS G12V), HCT116 (KRAS G13D), respectively. Then the CDS were cloned into pSin-EF2-Puro and processed for verification through Sanger sequencing to confirm their accuracy. For constructing the KRAS, P300 or GRAMD1A knockdown plasmids, short hairpin RNA (shRNA) sequences were designed and respectively cloned into lentiviral expression plasmid pLKO.1 (Addgene, #8453). pLV3-Mucin2(pig)-promoter-mCMV-CopGFP-Fluc-hRluc for dual luciferase reporter assay were obtained from MiaoLingBio (Wuhan, China), and the Mucin2 promoter region was removed to build the pLV3-mCMV-CopGFP-Fluc-hRluc vector plasmid. The GRAMD1A promoter sequence (sequence within 2000 bp before the transcription start site of GRAMD1A, NC_000019.10:34992727-34994726 Homo sapiens chromosome 19, GRCh38.p14 Primary Assembly) were synthesized by BGI Biotechnology (Beijing, China) and then inserted into the pLV3-mCMV-CopGFP-Fluc-hRluc dual luciferase reporter vector. pEZX-FR01 for dual luciferase reporter assay were obtained from GeneCopoeia (Guangzhou, China). The promoter region of HMGCR (sequence within 1000 bp before the transcription start site of HMGCR, NC_000005.10: 75336529-75337528 Homo sapiens chromosome 5, GRCh38.p14 Primary Assembly) was amplified by PCR and then inserted into pEZX-FR01 dual luciferase reporter vector. All plasmids were verified using sanger sequencing. For transient infection, plasmids were premixed with Lipofectamine® 3000 (Invitrogen), and then added into the corresponding cells. For stable infection, the indicated plasmids associated with the packaging plasmids (psPAX2 and pMD2.G; Addgene) were premixed with polyethylenimine (Polysciences, #24765-100) following manufacturer's instructions, and added into HEK293T cells to create lentivirus. Cells were cultured with the supernatant containing lentivirus and polybrene (Beyotime, #C0351) to transfection. Later, transfected cells were selected with puromycin (Beyotime, #ST551) or G418 (MP, # 0215878291) to obtain the stable transfection cell lines. Specific primers provided in Table S3 were used to construct plasmids.

## Dual luciferase reporter assay

Dual luciferase activities were examined utilizing the Dual Luciferase Reporter Gene Assay Kit (GeneCopoeia, #LF005) following manufacture’s manual. GRAMD1A promoter dual luciferase reporter plasmids were stably or transiently introduced into CRC cells that were treated with 10mM oxamate or both 10mM oxamate and 10mM Nala.

## Metabolomics

Cells were harvested and frozen in liquid nitrogen. To prepare the sample, cells were transferred into a 2 mL centrifuge tube and 100 mg glass bead and 1000 µL acetonitrile (ACN): methanol: H2O mixed solution (2:2:1, V/V/V) was added. Then, centrifuge tube containing the sample were vortexed, immersed in liquid nitrogen for rapid freezing, thawed at room temperature, and installed into the tissue grinder to grind for twice. Centrifuge tube was centrifuged for 10 min at 12,000 rpm and 4℃ to get the supernatant. The supernatant was concentrated, dried, added 300 µL acetonitrile:2-Amino-3-(2-chloro-phenyl)-propionic acid (4 ppm)

solution prepared with 0.1% formic acid (1:9, V / V) to redissolve, filtered by 0.22 µm membrane, and transfer into the detection bottle for LC-MS detection. The LC analysis was performed on a Vanquish UHPLC System (Thermo Fisher Scientific, USA). Chromatography was carried out with an ACQUITY UPLC ® HSS T3 (2.1× 100 mm, 1.8 µm) (Waters, Milford, MA, USA). Mass spectrometric detection of metabolites was performed on Orbitrap Exploris 120 (Thermo Fisher Scientific, USA) with ESI ion source. Simultaneous MS1 and MS/MS (Full MS-ddMS2 mode, data-dependent MS/MS) acquisition was used.

## Animal experiments

Animal experiments were performed in the Sixth Affiliated Hospital of Sun Yat-Sen University under the approval of the Institutional Animal Care and Use Committee (IACUC) at the Sixth Affiliated Hospital of Sun Yat-Sen University (IACUC-2022050701). Mice were randomly assigned to treatment and control groups using a computer-generated randomization sequence. In the mouse experiments, researchers were blinded during performing the treatments, data collection, and analyses. The sample size for mouse experiments was determined based on preliminary data and prior studies and power analysis (power = 0.8, alpha = 0.05) to detect a biologically meaningful effect size. The number of mice used was also minimized according to institutional ethical guidelines.

For the xenograft tumor model, 2 × 10^6^ HCT116 cells (si-Scramble, si-LDH, sh-Scramble and sh-GRAMD1A) or DLD1 cells (G13D/- sh-Scramble, G13D/+ sh-Scramble, +/- sh-Scramble, G13D/- sh- GRAMD1A, G13D/+ sh- GRAMD1A, +/- sh- GRAMD1A) were injected subcutaneously into BALB/c nude mice (female, 6-week-old). Tumor sizes were measured every two or five days utilizing digital calipers. Tumor volumes were calculated using the formula: Volume = (Longer diameter × Shorter diameter^2^) × 0.47. 20 or 25 days later, the mice were euthanized, xenograft tumors were weighed.

For colon orthotopic CRC mouse model, 2 × 10^6^ LOVO cells (psin-Vector and psin-GRAMD1A) were injected into the wall of the cecum in BALB/c nude mice (female, 6-week-old). After 9 weeks, all the mice were sacrificed and the intestines and livers were collected to assess the tumor burden. The organs were processed as cryosections and stained using H&E for histological assessment.

For the construction of CRC PDX model, tumor tissues from two CRC patients with mutant or wild-type KRAS were implanted into BALB/c nude mice (female, 6-week-old). When the tumor size reached 2 cm^3^, the tumors were divided into equal volume ~ 2 mm^3^ and were subcutaneously implanted into BALB/c nude mice (female, 6-week-old). After ten days, mice were randomized into DMSO group, GSK2837808A group and U18666A group. In GSK2837808A group and U18666A group, mice received GSK2837808A at a dose of 10mg/kg or U18666A at a dose of 20mg/kg by intraperitoneal injection every 2 days. All mice were sacrificed 5 weeks later and subcutaneous tumors were subjected to H&E and immunofluorescence analysis.

## Bioinformation analysis

For ChIP-seq, raw data was processed by Bowtie2, Samtools, Deeptools and MACS2 software to find the peaks. Peaks data was annotated by ChIPseeker package. For RNA sequencing, raw data was processed by Bowtie2, Samtools, Deeptools and HTseq software. Differentially expressed genes were analyzed by Limma package. Gene expression data and corresponding patient information for TCGA were obtained from cBioportal (<https://www.cbioportal.org/>).

# Supplementary tables

## Table S1 The primers for ChIP-qPCR

| Target | Forward primer | Reverse primer |
| --- | --- | --- |
| GRAMD1A promoter | AGCATTCTTGTGCCCCTACG | TCTTGTGCCCCTACGATGC |
|  |  |  |

## Table S2 The primers for RT--qPCR

| Gene | Forward primer | Reverse primer |
| --- | --- | --- |
| ACTB | GTCATTCCAAATATGAGATGCGT | GCTATCACCTCCCCTGTGTG |
| GRAMD1A | ACTGCTTGAAAAGGACCCCC | GCTCCTCCTTGTCCTCCTCT |
|  |  |  |

## Table S3 The primers for plasmid construction

| Name | Forward primer | Reverse primer |
| --- | --- | --- |
| psin-KRAS | CCCGGACGAATTCTTCGAAATGACTGAATATAAACTTGTGGTAGTTGGA | TGCGGATCACTAGTGCTAGCTTACATAATTACACACTTTGTCTTTGACTT |
| sh-KRAS | CCGGTAGCAAGAAGTTATGGAATTCCTCGAGGAATTCCATAACTTCTTGCTATTTTTG | AATTCAAAAATAGCAAGAAGTTATGGAATTCCTCGAGGAATTCCATAACTTCTTGCTA |
| sh-P300 | CCGGATACTCAGCCGGAGGATATTTCTCGAGAAATATCCTCCGGCTGAGTATTTTTTG | AATTCAAAAAATACTCAGCCGGAGGATATTTCTCGAGAAATATCCTCCGGCTGAGTAT |
| psin-GRAMD1A | CCCGGACGAATTCTTCGAAATGTTCGACACCACACCCCACTCTGGCCGG | TGCGGATCACTAGTGCTAGCTCAGGAAAAGCTGTCATCGGGCCGGGGCTG |
| GRAMD1A-sh1 | CCGGACGGAACCGAGATGCACTTTACTCGAGTAAAGTGCATCTCGGTTCCGTTTTTTG | AATTCAAAAAACGGAACCGAGATGCACTTTACTCGAGTAAAGTGCATCTCGGTTCCGT |
| GRAMD1A-sh2 | CCGGAGCGGCATTGAAGACTATTTCCTCGAGGAAATAGTCTTCAATGCCGCTTTTTTG | AATTCAAAAAAGCGGCATTGAAGACTATTTCCTCGAGGAAATAGTCTTCAATGCCGCT |
| pEZX-HMGCR | ACGCGTATTTAAATGTCGAAGAAGGAACGCACAGAAGACGCAGGAGAGG | ATGGTGGCGGATCCTCTAGCCTGCCGGGCCTCAAGGACATTCCTTGCAG |
| PLV3-GRAMD1A | TTCTTTCCGCCTCAGGCTAGACTCCCGAAAAAAAGCAAAG | ACTTCCTCTGCCCTCACCGGAGAGAGTCATGACCTTGCAG |
|  |  |  |

# Supplementary Figures


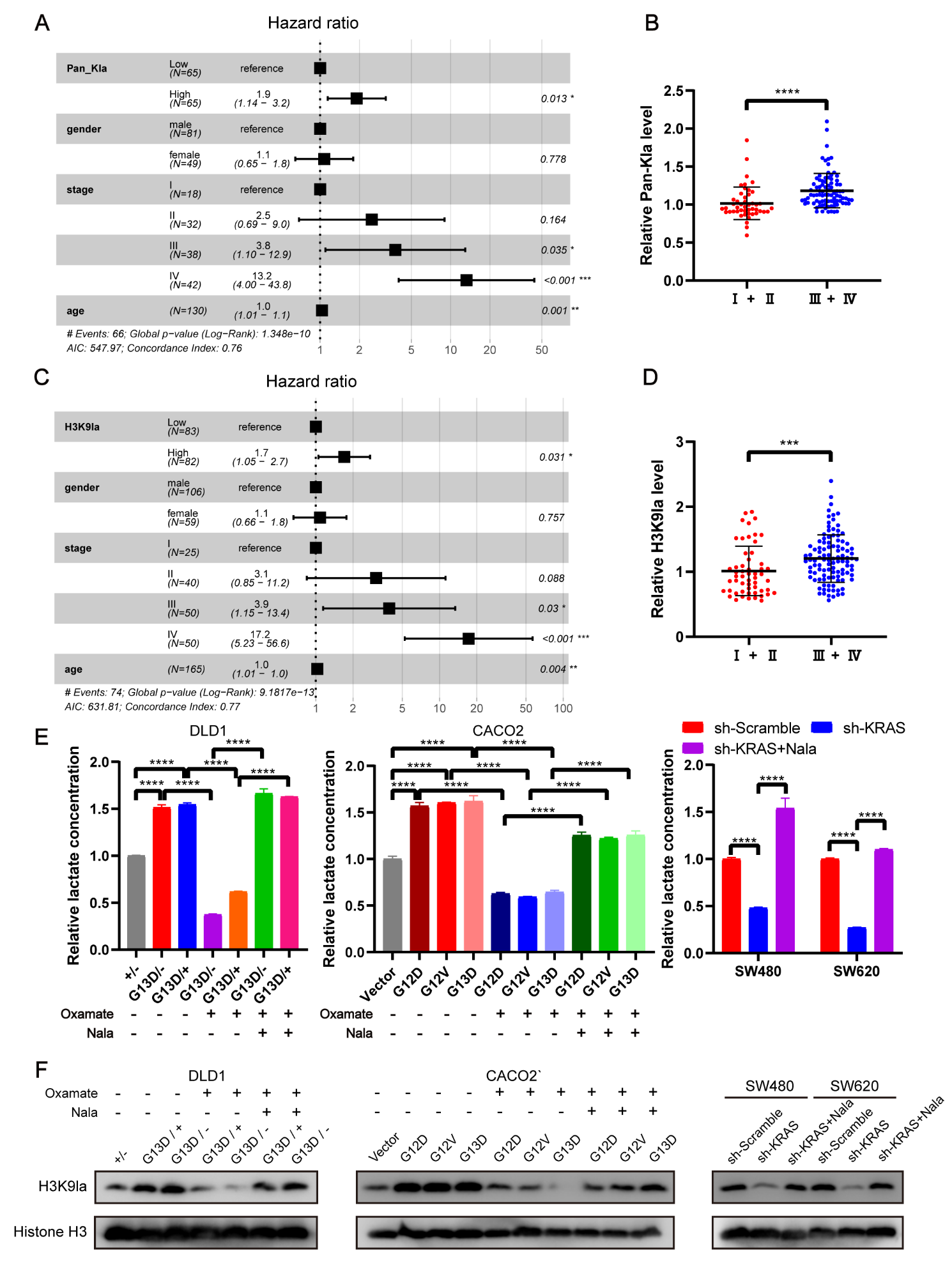


Fig.S1. KRAS mutant CRC exhibited elevated lactylation which was associated with poor patient survival. **(A, C)** Cox multivariate regression analysis to analyze the effects of **(A)** lactylation and **(C)** H3K9la on the survival of CRC patients. **(B, D)** Comparison of **(B)** Lactylation and **(D)** H3K9la level between patients with clinical stage I–II and those with clinical stage III–IV, detected by immunofluorescence. **(E-F)** **(E)** Intracellular lactate levels measured by a lactate colorimetric kit and **(F)** western blot of H3K9la levels, in DLD1 cell lines harboring wild-type KRAS (+ / -), G13D mutant KRAS (G13D / -) or both alleles (G13D / +) of KRAS (left), CACO2 cells stably transfected with vector and cDNA encoding the KRAS^G12D^ , KRAS^G12V^ and KRAS^G13D^ mutations (middle), and SW480 and SW620 cells transfected with KRAS-specific shRNA (right). Cells were treated with 20 mM oxamate or 10 mM Nala or both for 24 hours. n = 3. Values are presented as mean ± SD. *** *p* < 0.001, **** *p* < 0.0001, determined by Mann-Whitney U test (B, D) and one-way ANOVA (E).


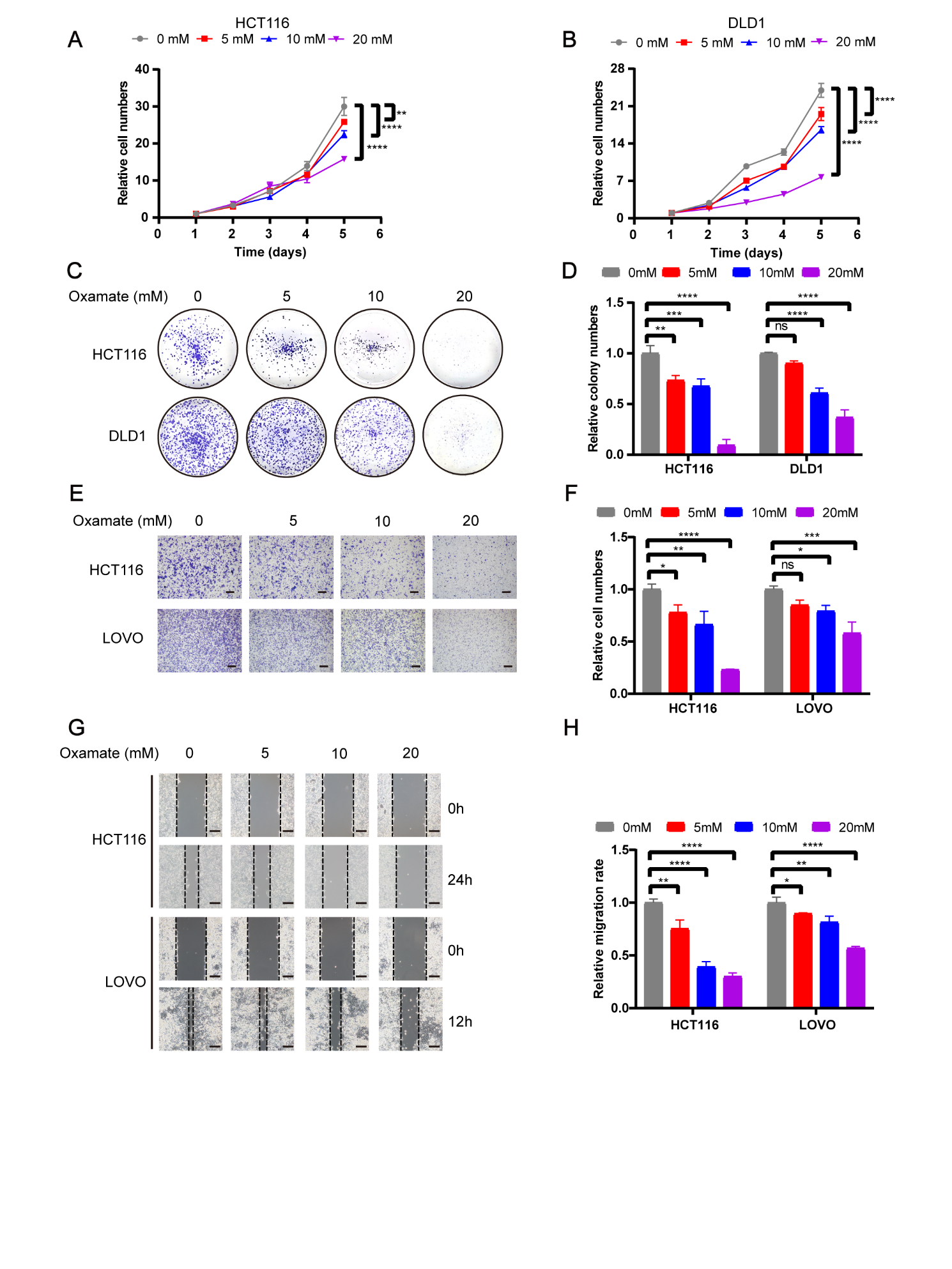


Fig.S2. Inhibition of H3K9la and lactylation by oxamate suppressed proliferation and migration of CRC. **(A-B)** Proliferation of **(A)** HCT116 and **(B)** DLD1 cells cultured in different concentration of oxamate analyzed by CCK8 assay. n = 4. **(C, D)** Tumor growth of HCT116 and DLD1 cells cultured in different concentration of oxamate evaluated by **(C)** colony formation assay and **(D)** statistical analysis. n = 3. **(E, F)** Migration of HCT116 and LOVO cells cultured in different concentration of oxamate evaluated by **(E)** transwell assay and **(F)** statistical analysis. n = 3. Scale bar: 200 μm. **(G, H)** Migration of HCT116 and LOVO cells cultured in different concentration of oxamate evaluated by **(G)** wound healing assay and **(H)** statistical analysis. n = 3. Scale bar: 100 μm. Values are presented as mean ± SD. * *p* < 0.05, ** *p* < 0.01, *** *p* < 0.001, **** *p* < 0.0001, ns *p* > 0.05, determined by one-way ANOVA (A-B, D ,F ,H).
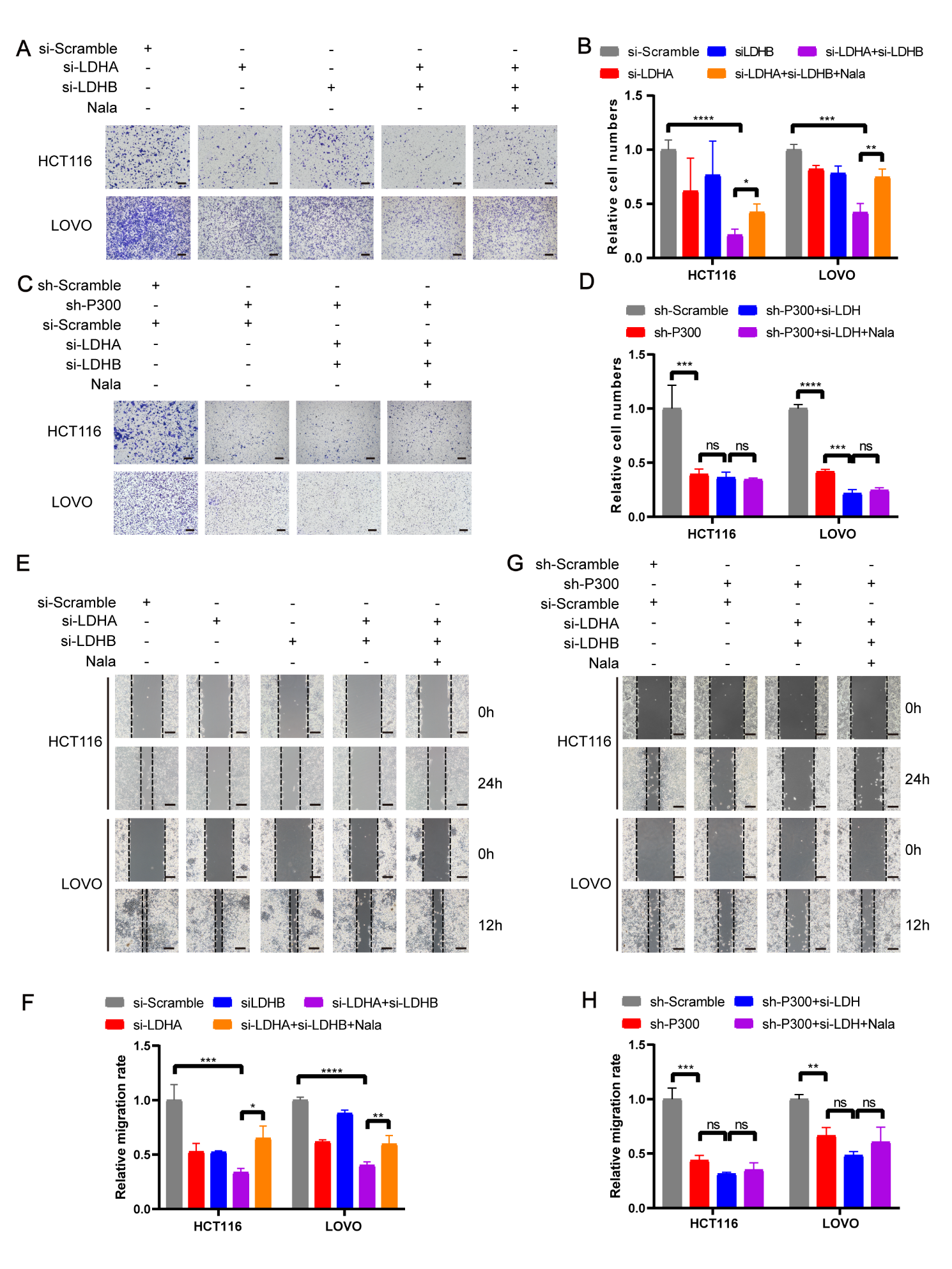


Fig.S3. Inhibition of H3K9la and lactylation by silencing LDH suppressed migration of CRC. **(A-B)** Migration of HCT116 and DLD1 cells after silencing LDHA, LDHB or both evaluated by **(A)** Transwell assay and **(B)** statistical analysis. n = 3. Scale bar: 200 μm. **(C-D)** Migration of P300 knock-down HCT116 and DLD1 cells after silencing both LDHA and LDHB evaluated by **(C)** transwell assay and **(D)** statistical analysis. n = 3. Scale bar: 200 μm. **(E-F)** Migration of HCT116 and DLD1 cells after silencing LDHA, LDHB or both evaluated by **(E)** wound healing assay and **(F)** statistical analysis. n = 3. Scale bar: 100 μm. **(G-H)** Migration of P300 knock-down HCT116 and DLD1 cells after silencing both LDHA and LDHB evaluated by **(G)** Wound healing assay and **(H)** statistical analysis. n = 3. Scale bar: 100 μm. Values are presented as mean ± SD. * *p* < 0.05, ** *p* < 0.01, *** *p* < 0.001, **** *p* < 0.0001, ns *p* > 0.05, determined by one-way ANOVA (B, D ,F ,H).


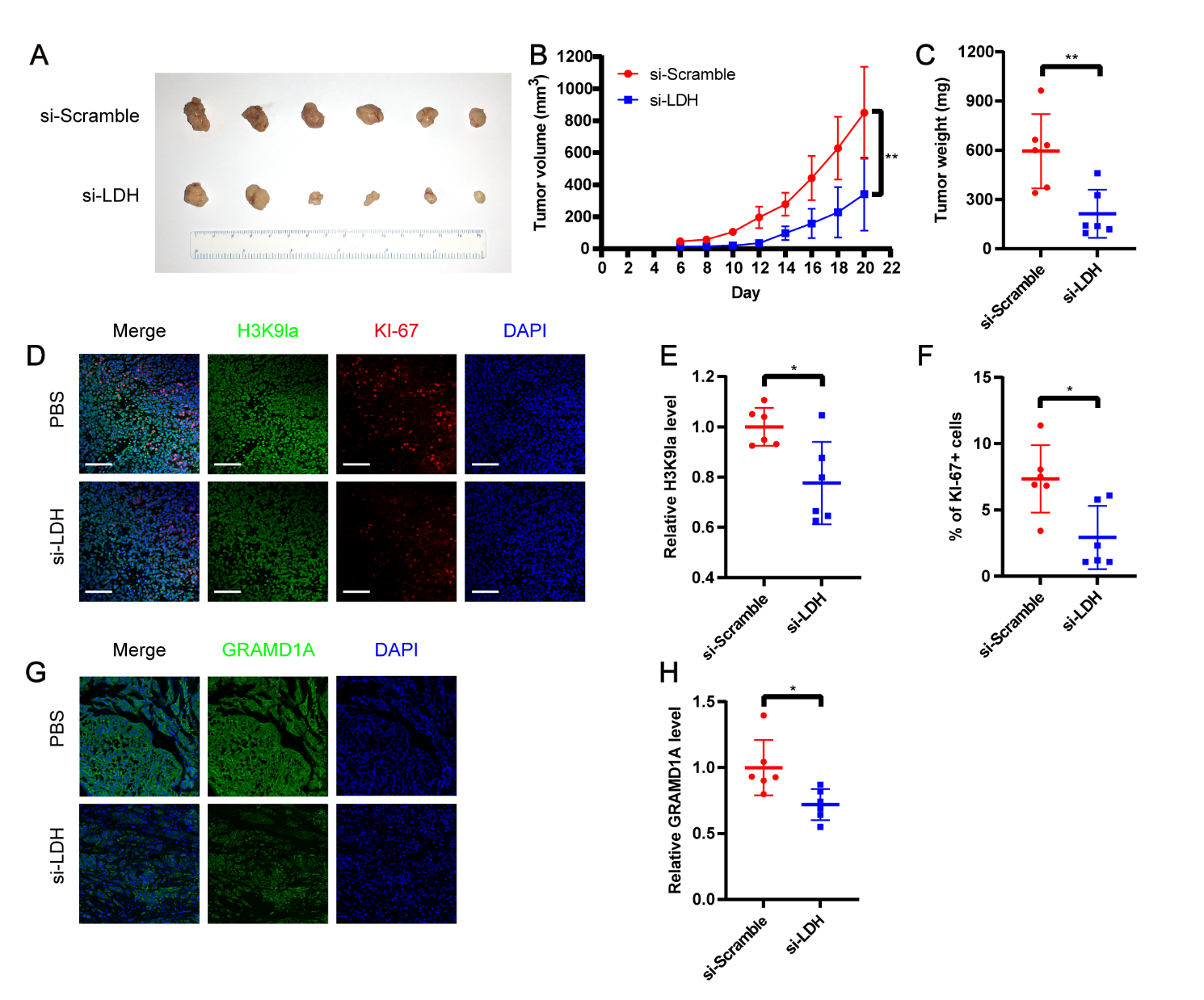


Fig.S4. Inhibition of H3K9la by silencing LDH suppressed growth of CRC in vivo. **(A-C) (A)** General picture, **(B)** volume and **(C)** weight of subcutaneous xenografts of HCT116 cells inhibited lactylation by LDHA and LDHB silencing. **(D)** Immunofluorescence staining of lactylation levels and Ki-67 expression in subcutaneous xenografts of HCT116 cells inhibited lactylation by LDHA and LDHB silencing. Scale bar: 100 μm. **(E-F)** Statistical immunofluorescence results of **(E)** lactyation levels and **(F)** Ki-67 expression in subcutaneous xenografts. **(G-H) (G)** Immunofluorescence staining and **(H)** statistic of GRAMD1A expression in subcutaneous xenografts of HCT116 cells inhibited lactylation by LDHA and LDHB silencing. Scale bar: 100 μm. Values are presented as mean ± SD. ** *p* < 0.01, **** *p* < 0.0001, determined by two-tailed Student's t-test (B-C, E-F, H).


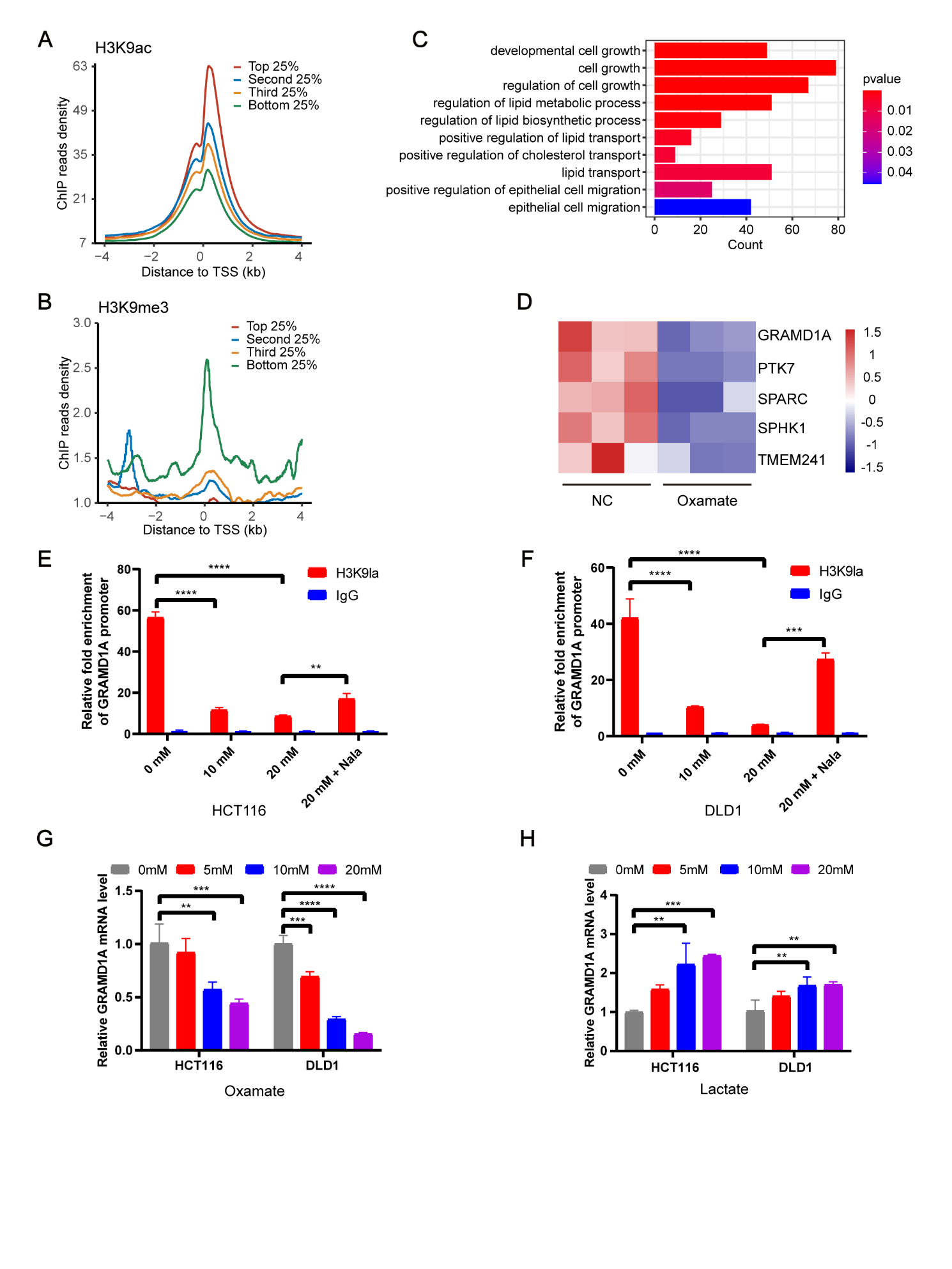


Fig.S5. H3K9la promoted GRAMD1A transcription in CRC. **(A-B) (A)** H3K9ac correlated with steady-state mRNA levels. **(B)** H3K9me3 negatively correlated with steady-state mRNA levels. The average ChIP signal intensity (read count per million mapped reads) for indicated antibodies is shown for genes with different expression levels (the top 25%, the second 25%, the third 25%, and the bottom 25% of RNA-seq counts). **(C)** Gene ontology (GO) enrichment analysis of down-regulated genes detected by RNA-seq after oxamate treatment in HCT116 cells **(D)** Heatmap of mRNA level after oxamate treatment. **(E-F)** H3K9la level at GRAMD1A promoter region by ChIP-qPCR assay in **(E)** HCT116 cells and **(F)** DLD1 cells after oxamate and 10 mM Nala treatment. n=3. **(G-H)** GRAMD1A mRNA level by qPCR assay in HCT116 cells and DLD1 cells after **(G)** oxamate and **(H)** Nala treatment. n=3. Values are presented as mean ± SD. ** *p* < 0.01, *** *p* < 0.001, **** *p* < 0.0001, determined by one-way ANOVA (E-H).


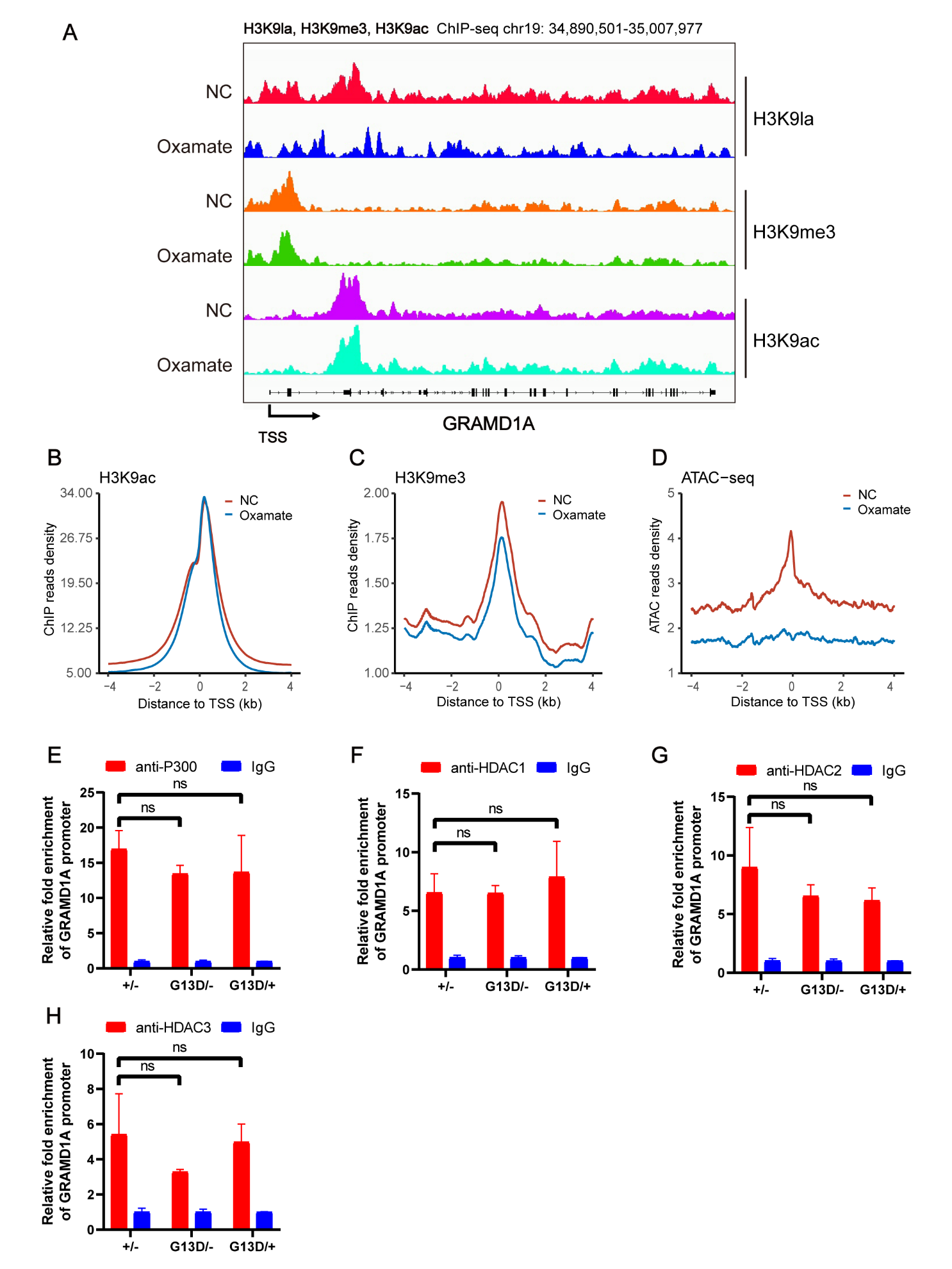


Fig.S6. H3K9la increased chromatin accessibility in CRC. **(A)** IGV tracks for GRAMD1A from H3K9la, H3K9ac, and H3K9me3 ChIP-seq analysis. **(B-C)** Distribution and level of **(B)** H3K9ac and **(C)** H3K9me3 sites relative to translation start site (TSS) after LDH inhibitor oxamate treatment. **(D)** Distribution and level of chromatin accessible sites relative to translation start site (TSS) after LDH inhibitor oxamate treatment. **(E-H)** The binding of the lactylation writer P300 **(E)** and the lactylation erasers HDAC1 **(F)**, HDAC2 **(G)**, HDAC3 **(H)** to the promoter region of GRAMD1A by ChIP-qPCR assay. n=3. Values are presented as mean ± SD. ns *p* > 0.05, determined by Welch’s ANOVA (E-H).


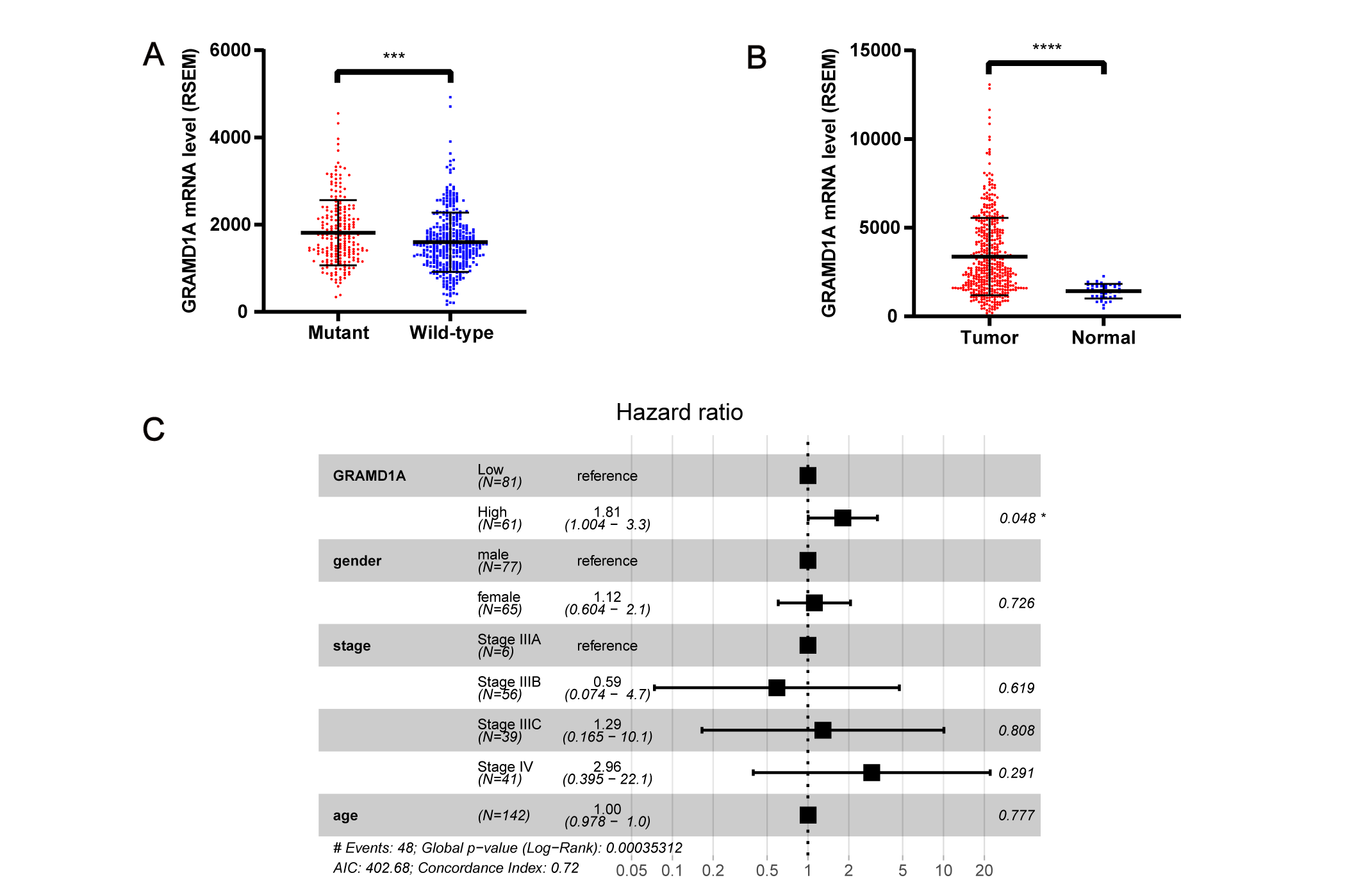


Fig.S7. Elevated GRAMD1A expression in KRAS mutant CRC. **(A-B)** GRAMD1A mRNA level in **(A)** CRC and normal colon tissues, and **(B)** KRAS mutant and wild-type CRC tissues from TCGA datasets. **(C)** Cox multivariate regression analysis to analyze the effects GRAMD1A on the survival of CRC patients with advanced stages. Values are presented as mean ± SD. * *p* < 0.05, *** *p* < 0.001, **** *p* < 0.0001, determined by two-tailed Student's t-test (A-B).


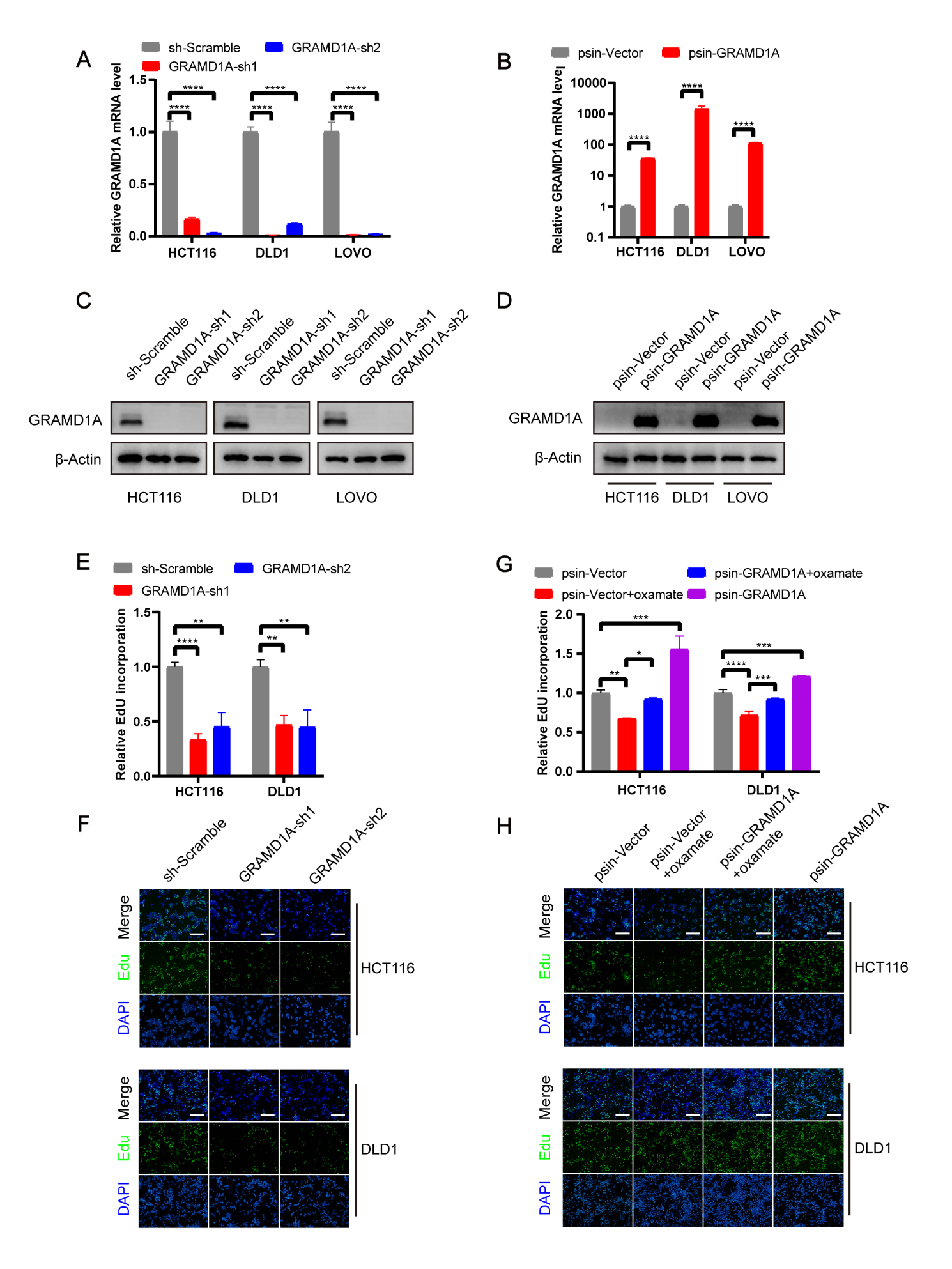


Fig.S8. GRAMD1A promoted proliferation of CRC. **(A-B)** GRAMD1A mRNA levels determined by qPCR in HCT116, DLD1 and LOVO cells after **(A)** knocking down and **(B)** overexpression. n=3. **(C-D)** GRAMD1A protein levels determined by western blot in HCT116, DLD1 and LOVO cells after **(C)** knocking down and **(D)** overexpression. **(E-H)** Proliferation of HCT116 and DLD1 cells after **(E-F)** knocking down and **(G-H)** overexpressing GRAMD1A by **(F, H)** Edu incorporation assay and **(E, G)** statistical analysis. n=3. Values are presented as mean ± SD. * *p* < 0.05, ** *p* < 0.01, *** *p* < 0.001, **** *p* < 0.0001, determined by one-way ANOVA (A, E, G) and two-tailed Student's t-test (B).


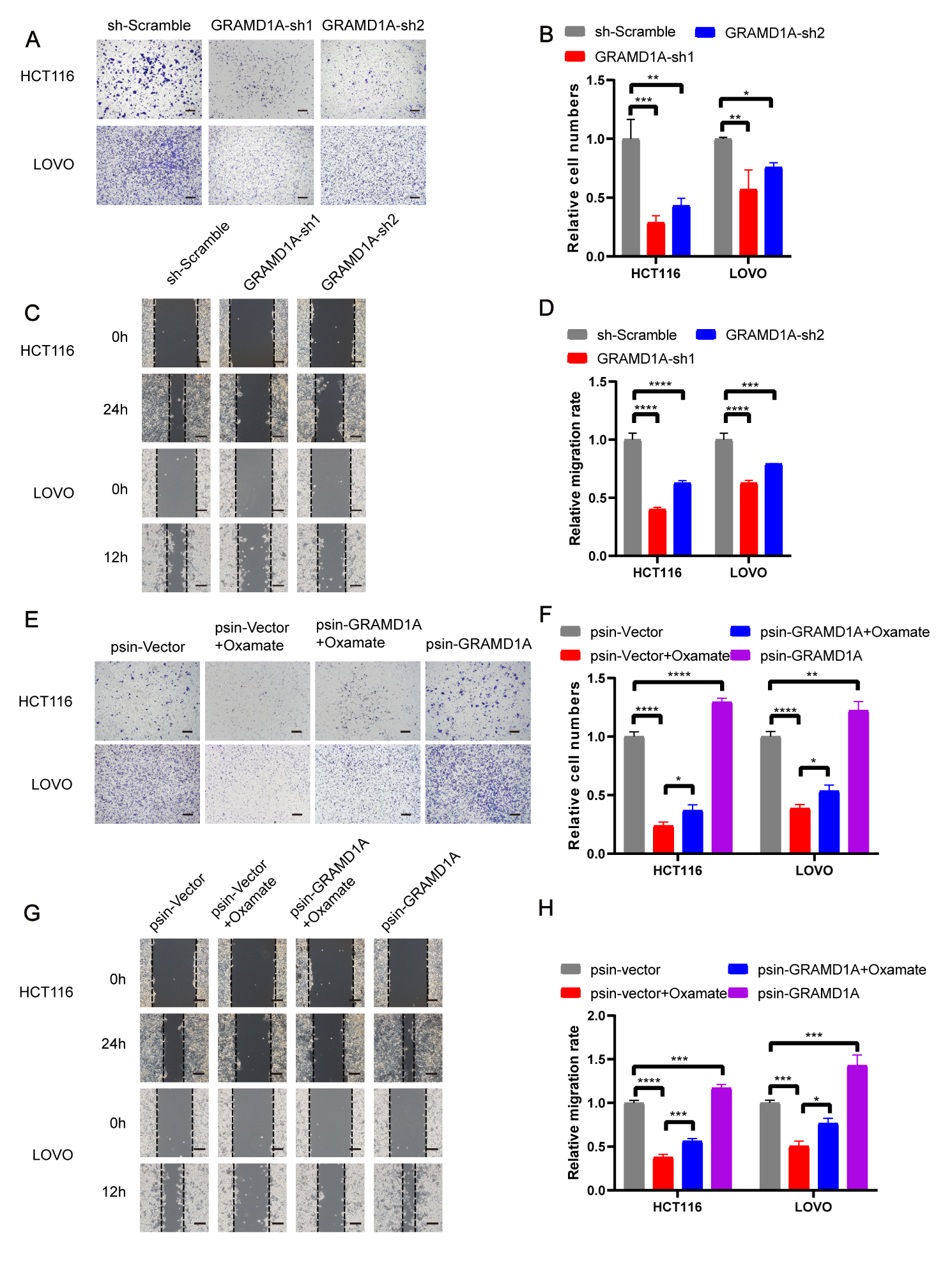


Fig.S9. GRAMD1A promoted migration of CRC. **(A-B)** Migration of HCT116 and DLD1 cells after knocking down GRAMD1A evaluated by **(A)** transwell assay and **(B)** statistical analysis. n = 3. Scale bar: 200 μm. **(C-D)** Migration of HCT116 and DLD1 cells after knocking down GRAMD1A evaluated by **(C)** wound healing assay and **(D)** statistical analysis. n = 3. Scale bar: 100 μm. **(E-F)** Migration of HCT116 and DLD1 cells after GRAMD1A overexpression with or without oxamate treatment evaluated by **(E)** transwell assay and **(F)** statistical analysis. n = 3. Scale bar: 200 μm. **(G-H)** Migration of HCT116 and DLD1 cells after GRAMD1A overexpression with or without oxamate treatment evaluated by **(G)** wound healing assay and **(H)** statistical analysis. n = 3. Scale bar: 100 μm. Values are presented as mean ± SD. *p < 0.05, ** *p* < 0.01, *** *p* < 0.001, **** *p* < 0.0001, determined by one-way ANOVA (B, D ,F ,H).


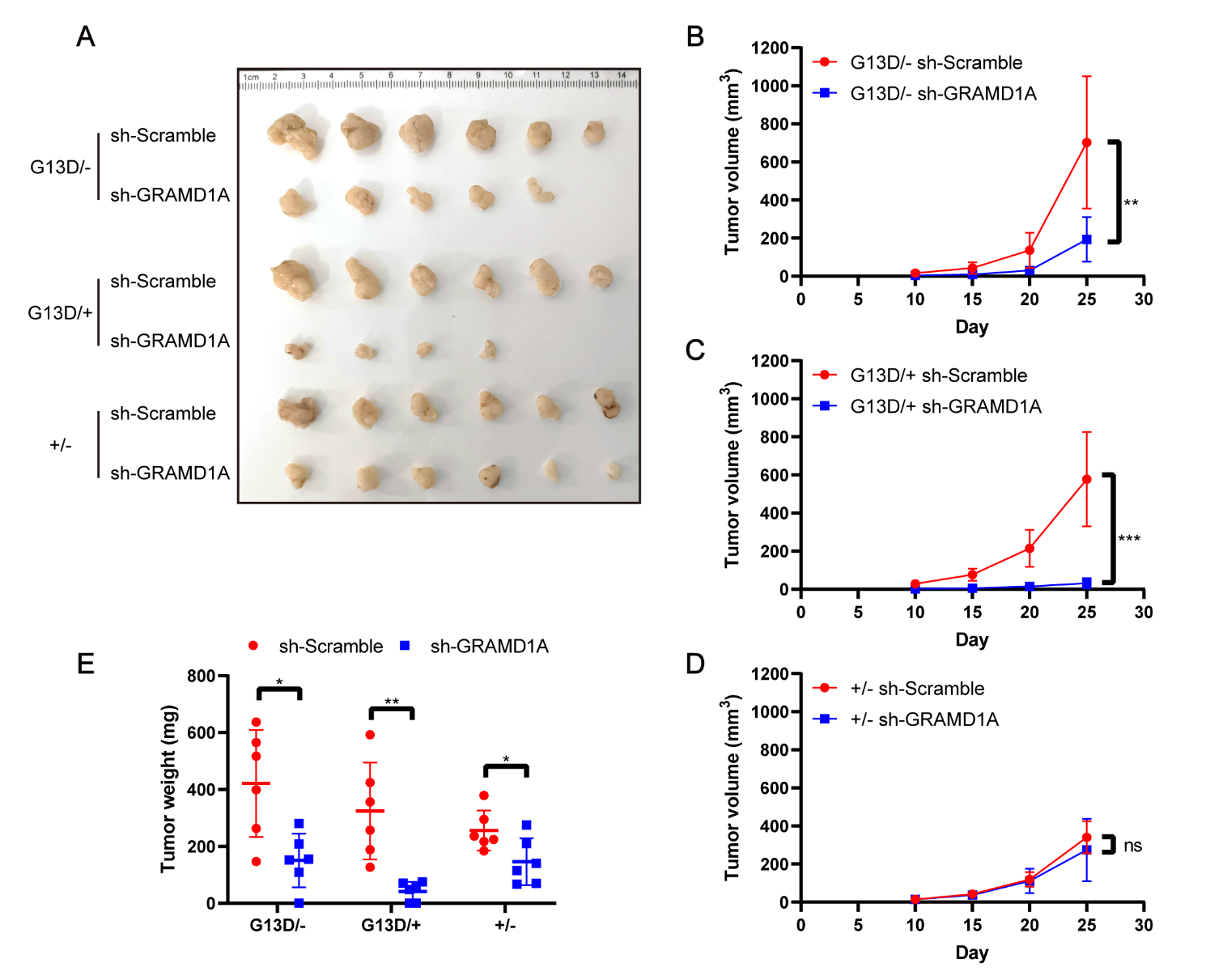


Fig.S10. GRAMD1A promoted growth of CRC in vivo. **(A)** Representative images of gross inspection of subcutaneous xenografts. **(B-D)** Statistical analysis of volumes of subcutaneous tumors from DLD1 cells harboring **(B)** mutant (G13D/-), **(D)** wild-type (+/-) or **(C)** both KRAS alleles (G13D/+). **(E)** Statistical analysis of weights of subcutaneous tumors. Values are presented as mean ± SD. ns *p* > 0.05, * *p* < 0.05, ** *p* < 0.01, *** *p* < 0.001, determined by two-tailed Welch’s t-test (B-D) and two-tailed Student's t-test (E).


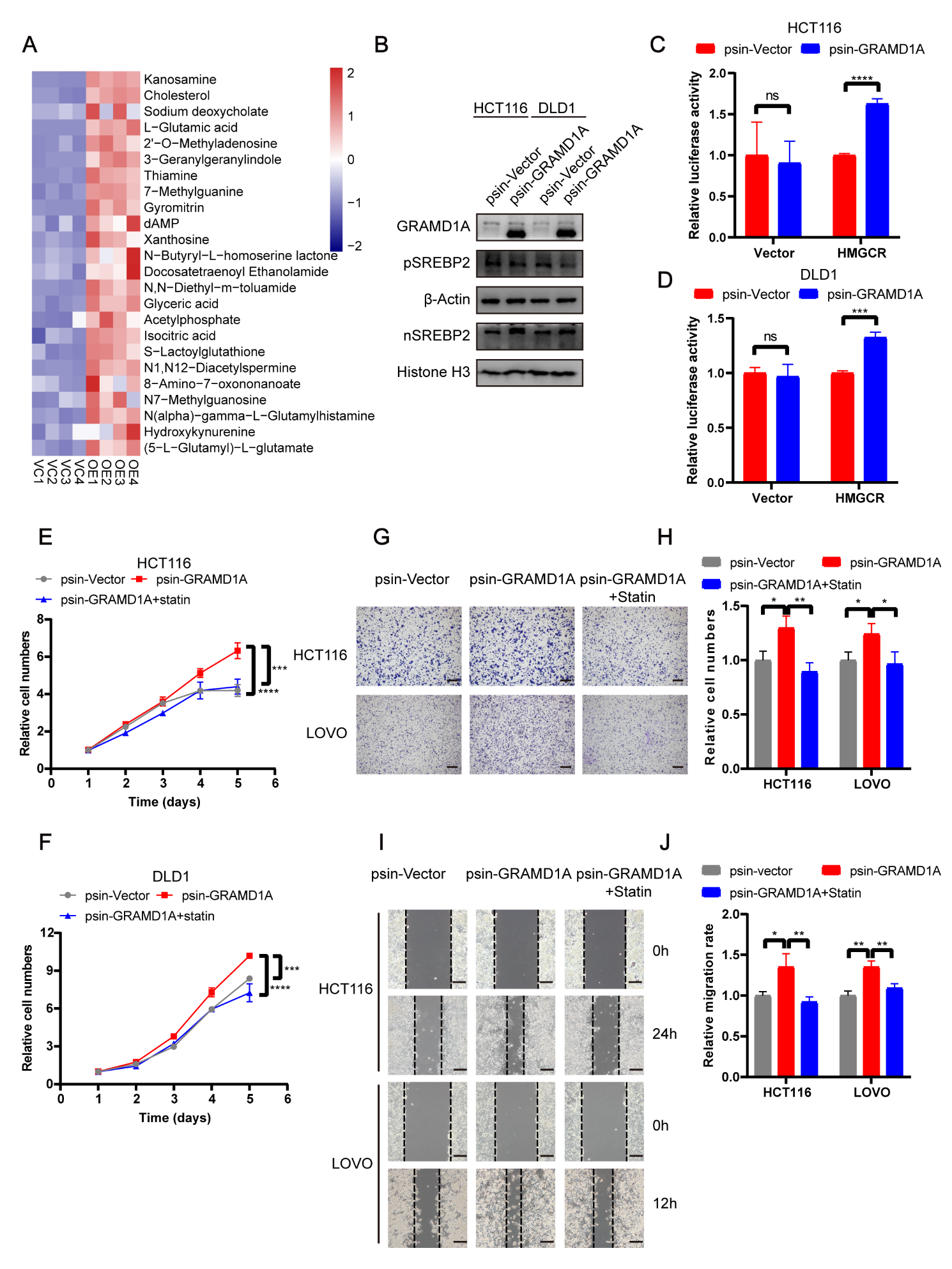


Fig.S11. GRAMD1A promoted CRC proliferation and migration through facilitating cholesterol metabolism. **(A)** Heatmap of upregulated metabolites after overexpression of GRAMD1A in HCT116 cells determined by metabolomics. **(B)** Protein levels of precursor form of SREBP2 (p-SREBP2) and nuclear form of SREBP2 (n-SREBP2) determined by western blot after GRAMD1A overexpression in HCT116 and DLD1 cells. **(C–D)** Relative luciferase activity in vector and GRAMD1A overexpressing **(C)** HCT116 and **(D)** DLD1 cells, transfected with a dual-luciferase reporter plasmid containing the promoter (sequence within 1000 bp before the transcription start site) of HMGCR or control vector. n=3. **(E-F)** Proliferation of **(E)** HCT116 and **(F)** DLD1 cells overexpressing GRAMD1A treated with 5 μM Simvastatin analyzed by CCK8 assay. n=4. **(G-H)** Migration of HCT116 and DLD1 cells after GRAMD1A overexpression treated with 10 μM Simvastatin analyzed by **(G)** transwell assay and **(H)** statistical analysis. n = 3. Scale bar: 200 μm. **(I-J)** Migration of HCT116 and DLD1 cells after GRAMD1A overexpression treated with 10 μM Simvastatin analyzed by **(I)** wound healing assay and **(J)** statistical analysis. n = 3. Scale bar: 100 μm. Values are presented as mean ± SD. ns > 0.05, * *p* < 0.05, ** *p* < 0.01, *** *p* < 0.001, **** *p* < 0.0001, determined by one-way ANOVA (E, F ,H ,J) and two-tailed Student's t-test (C, D).
