# Supplementary material for: KRAS mutation increases histone H3 lysine 9 lactylation (H3K9la) to promote colorectal cancer progression by facilitating cholesterol transporter GRAMD1A expression
Source: Cell Death Differ. 2025 Jul 24;32(12):2225–38. doi: 10.1038/s41418-025-01533-4 (PMC12669710; doi:10.1038/s41418-025-01533-4)

Figure 1 E

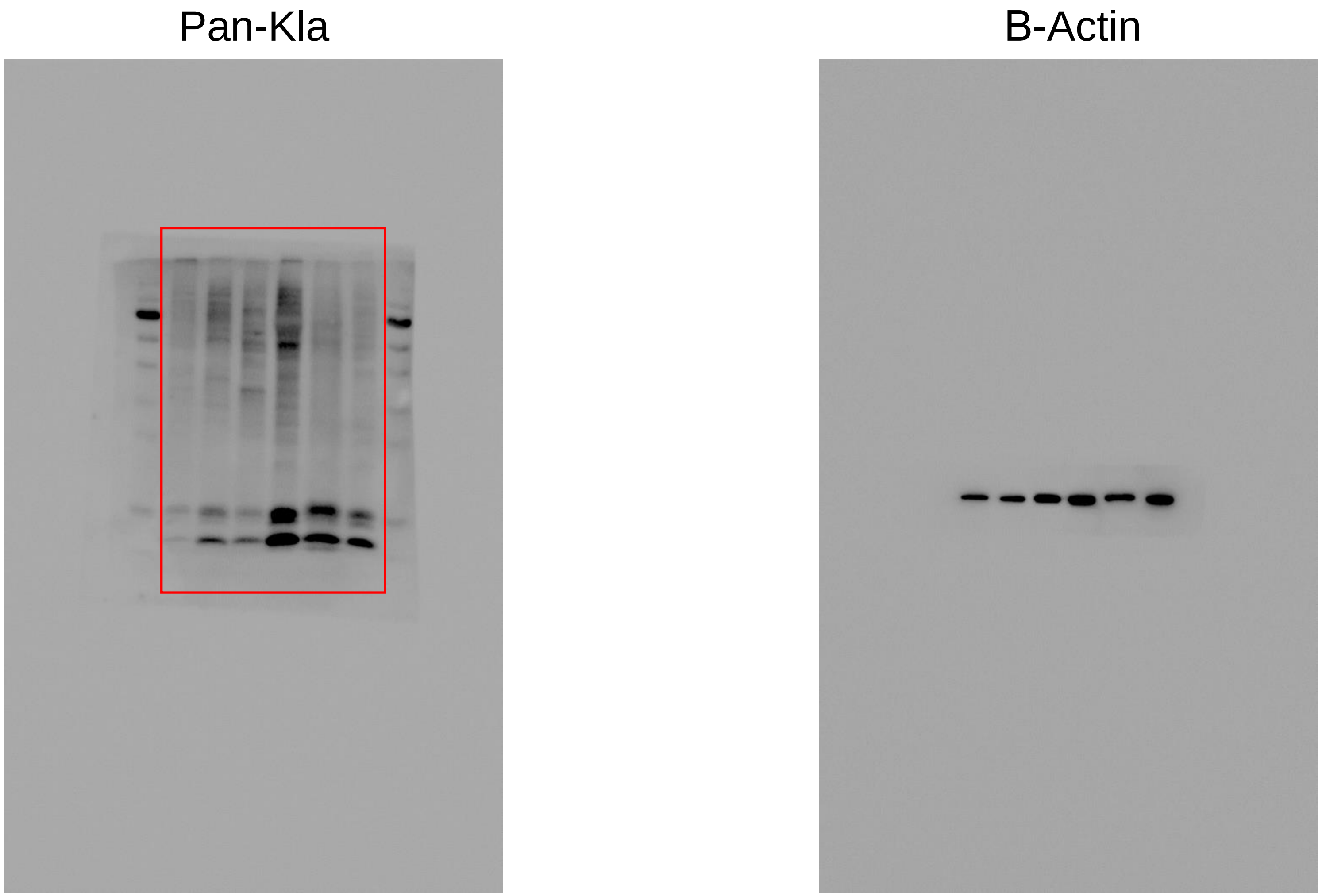

Figure 1 G

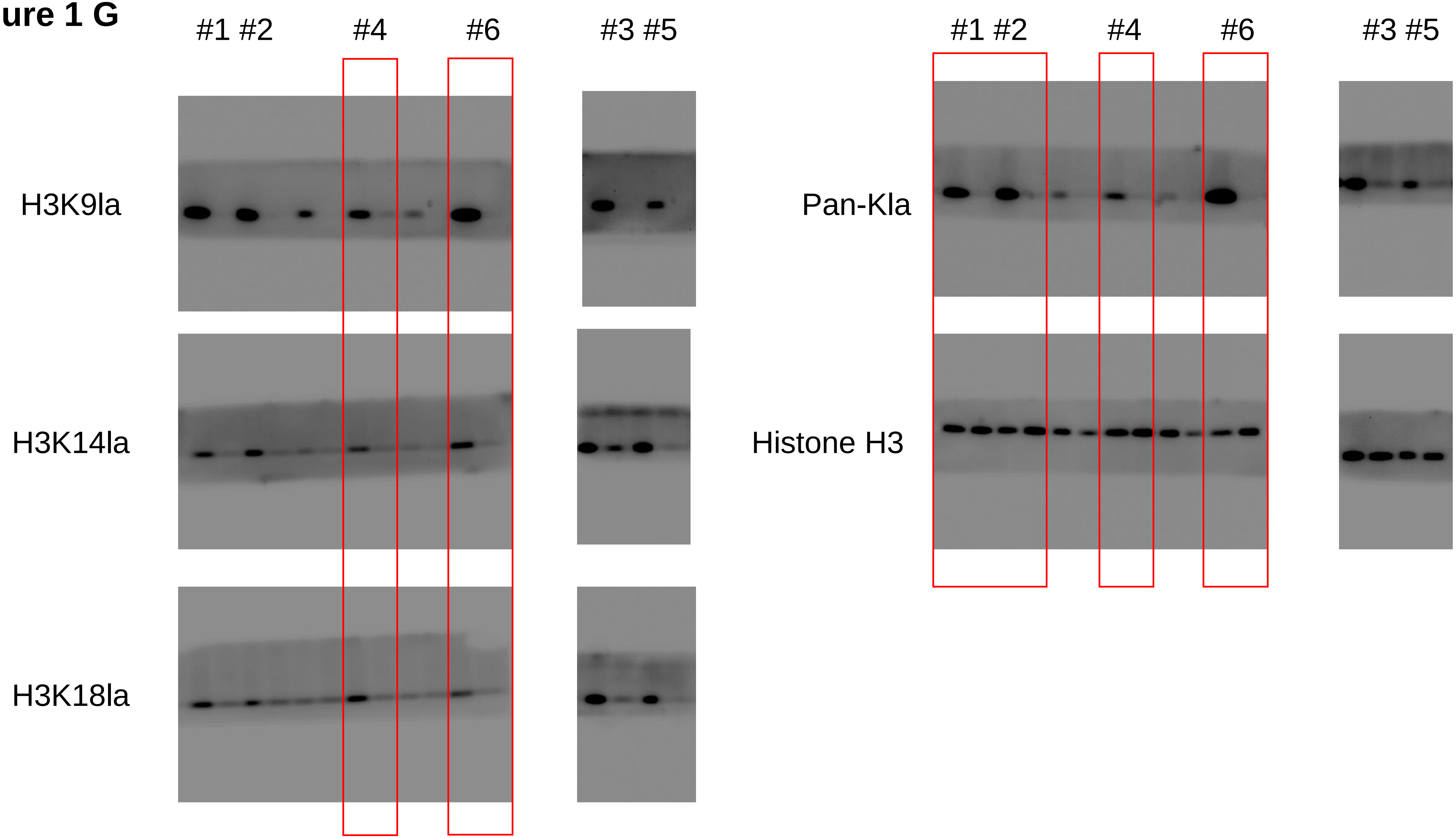

Figure 1 G

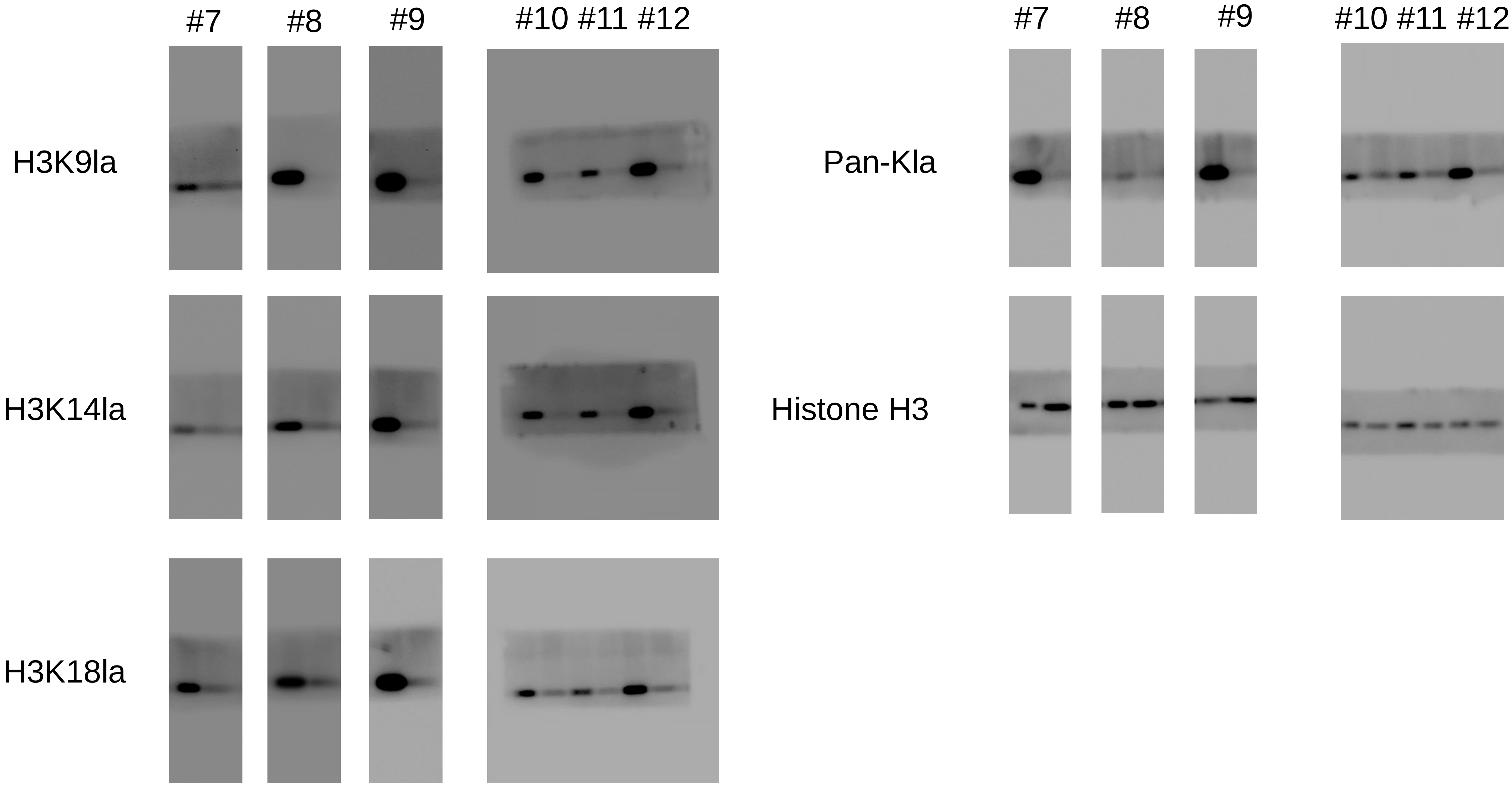

#7
#8
#9
#10 #11 #12
H3K9la
H3K14la
H3K18la
#7
#8
#9
#10 #11 #12
Pan-Kla
Histone H3

Figure 1 G

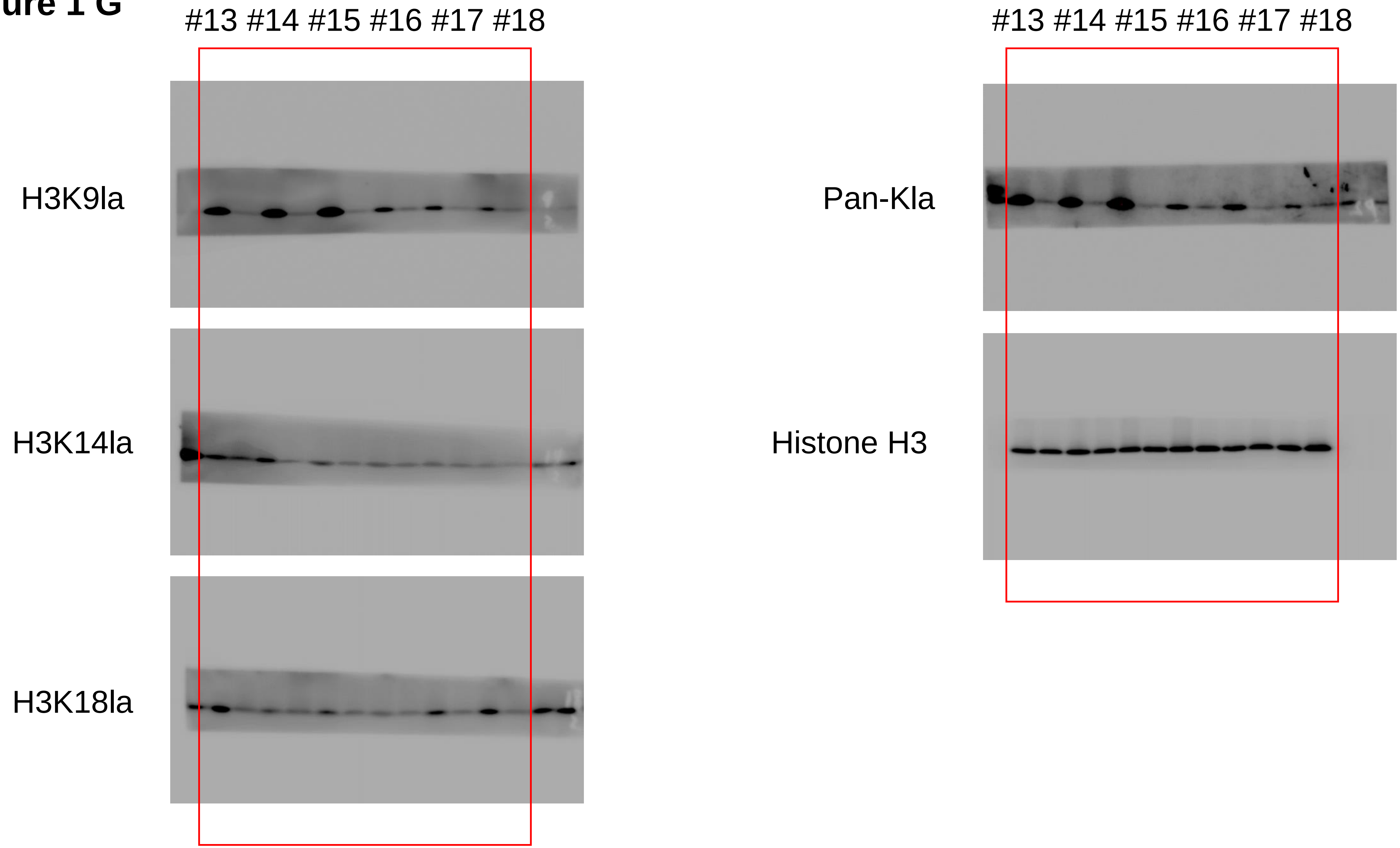
#13 #14 #15 #16 #17 #18
H3K9la
H3K14la
H3K18la
#13 #14 #15 #16 #17 #18
Pan-Kla
Histone H3

Figure 2 B

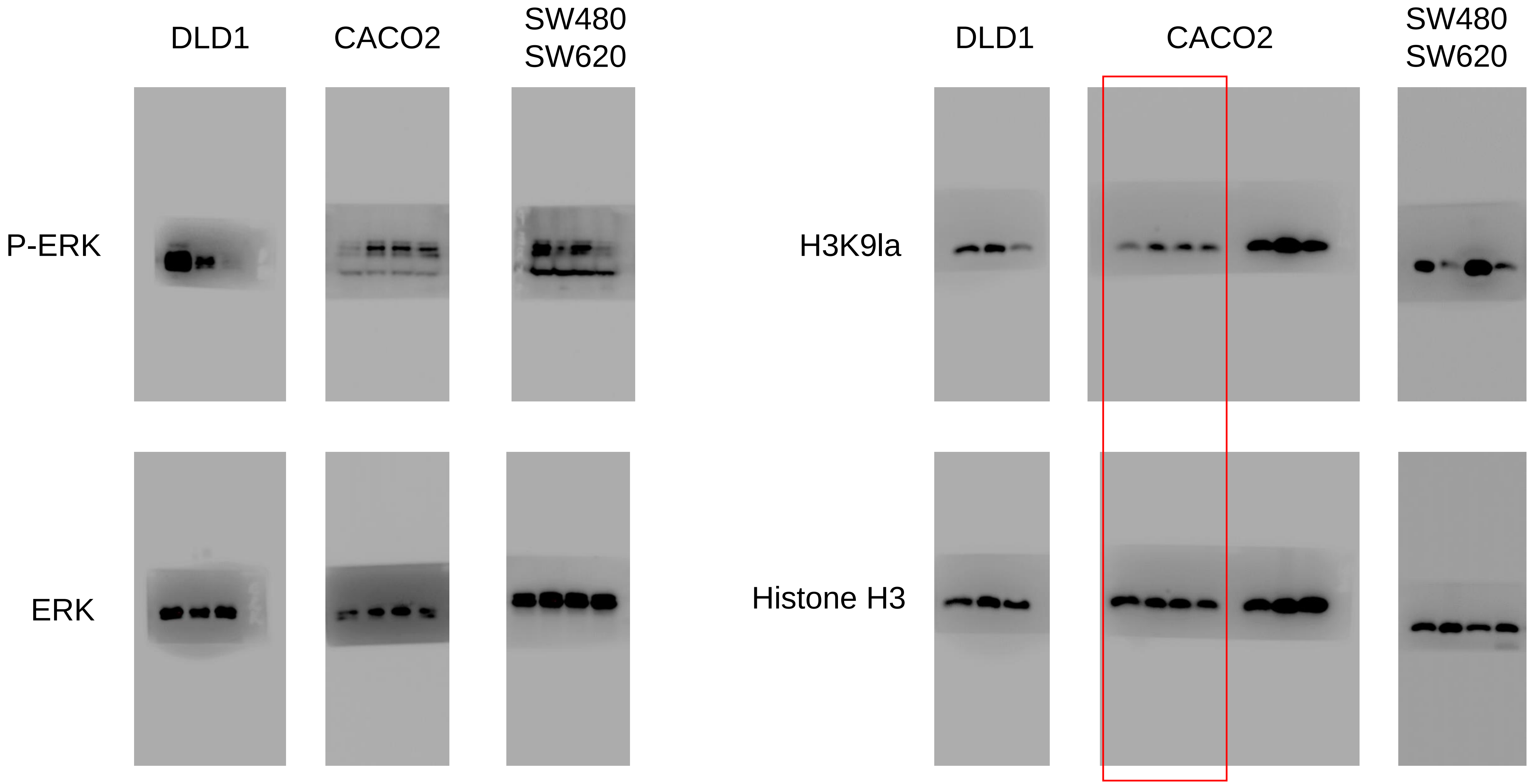
DLD1
CACO2
SW480
SW620
P-ERK
ERK
DLD1
CACO2
SW480
SW620
H3K9la
Histone H3

Figure 2 B

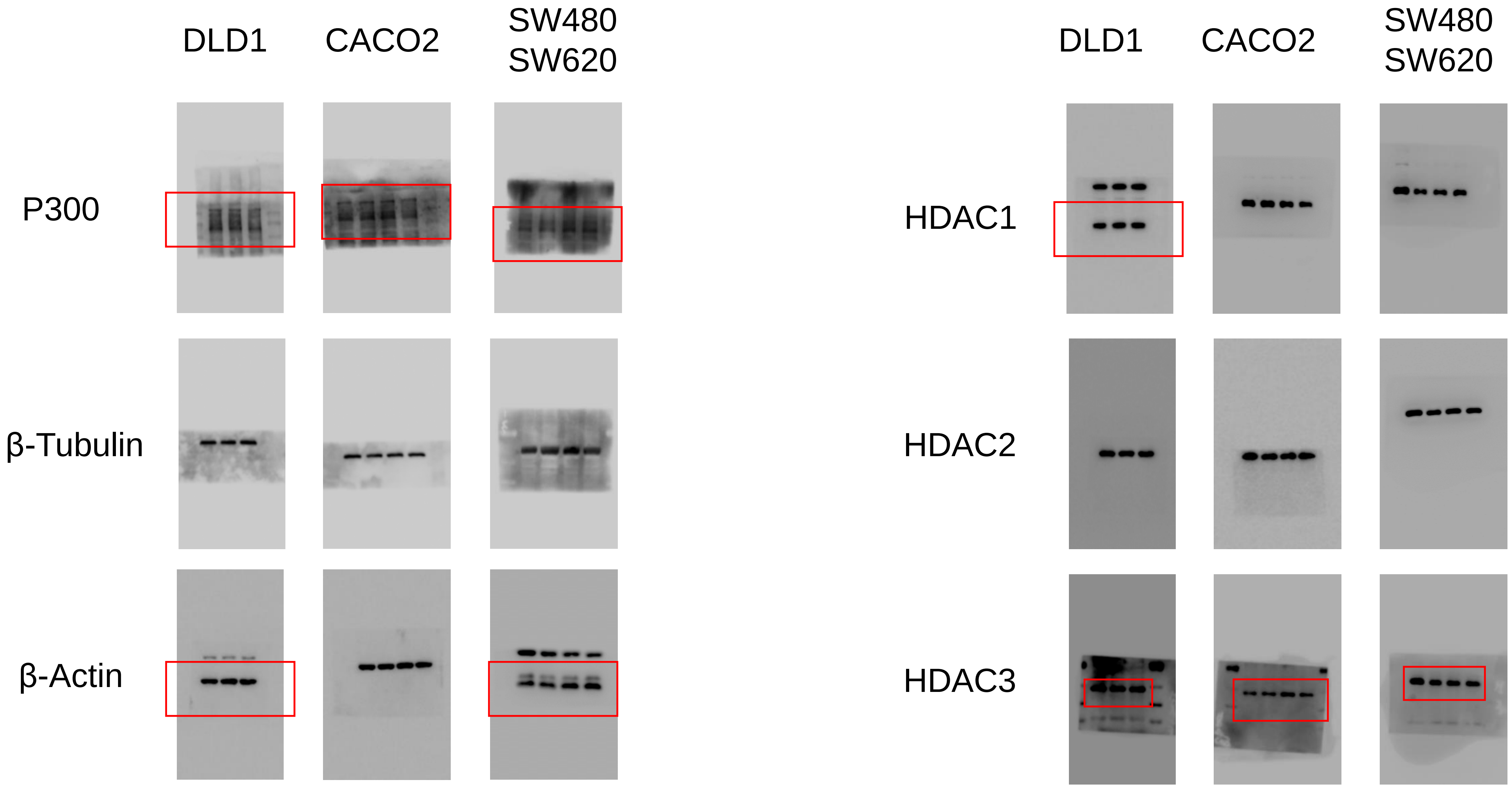

DLD1
CACO2
SW480
SW620
P300
β-Tubulin
β-Actin
DLD1
CACO2
SW480
SW620
HDAC1
HDAC2
HDAC3

Figure 2 I

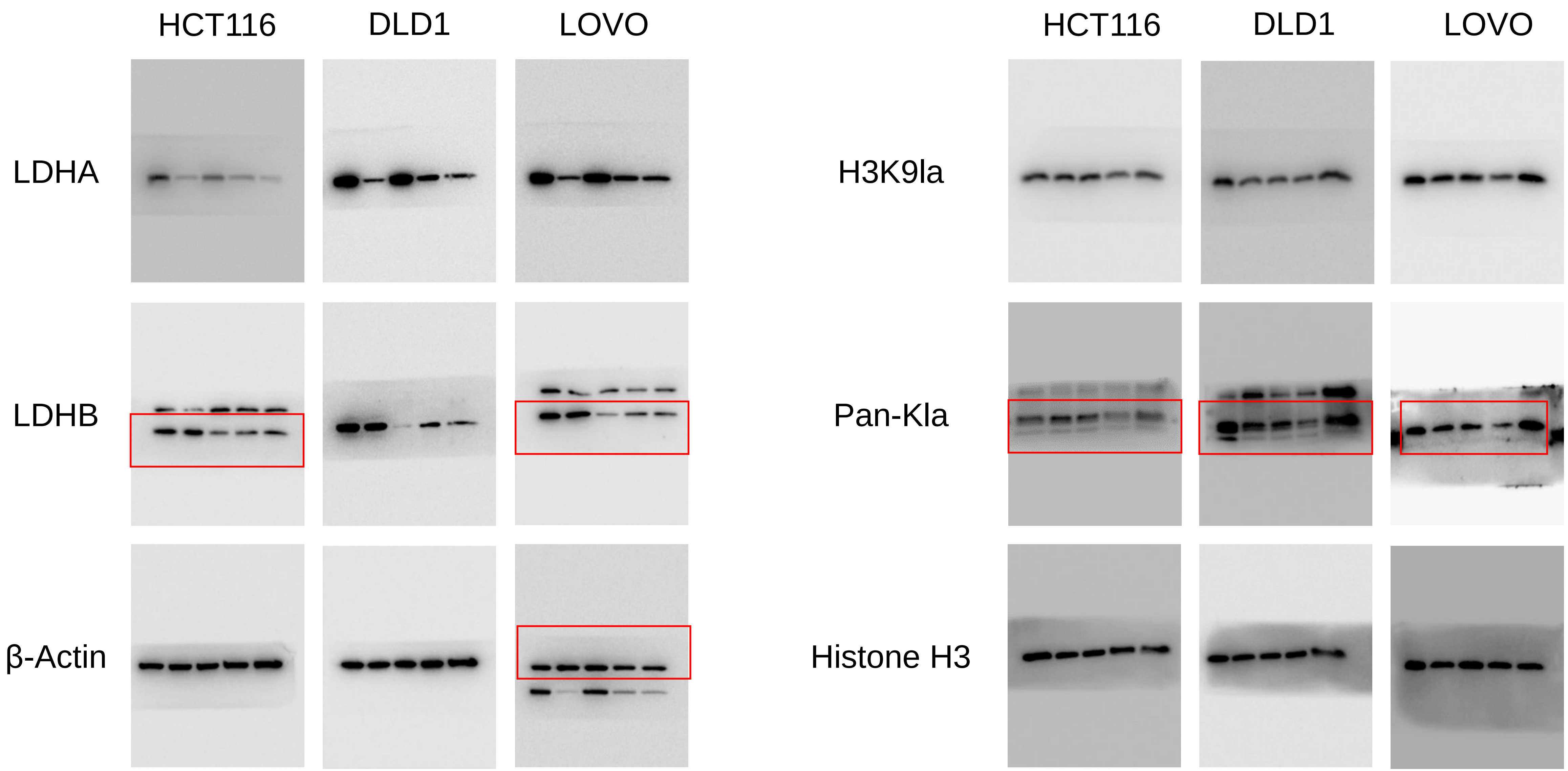
HCT116
DLD1
LOVO
LDHA
LDHB
β-Actin
HCT116
DLD1
LOVO
H3K9la
Pan-Kla
Histone H3

Figure 2 J

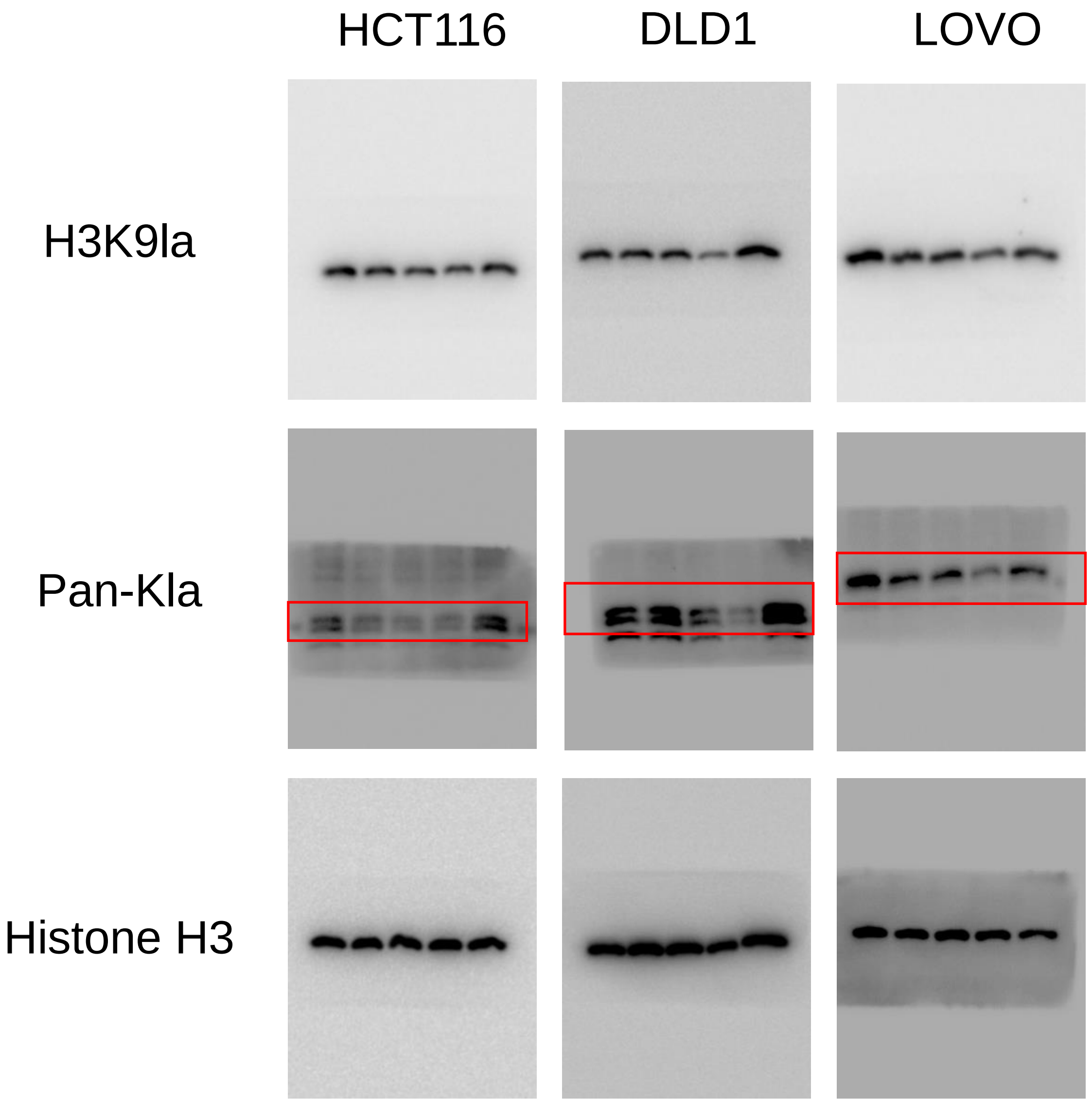

Figure 3 G

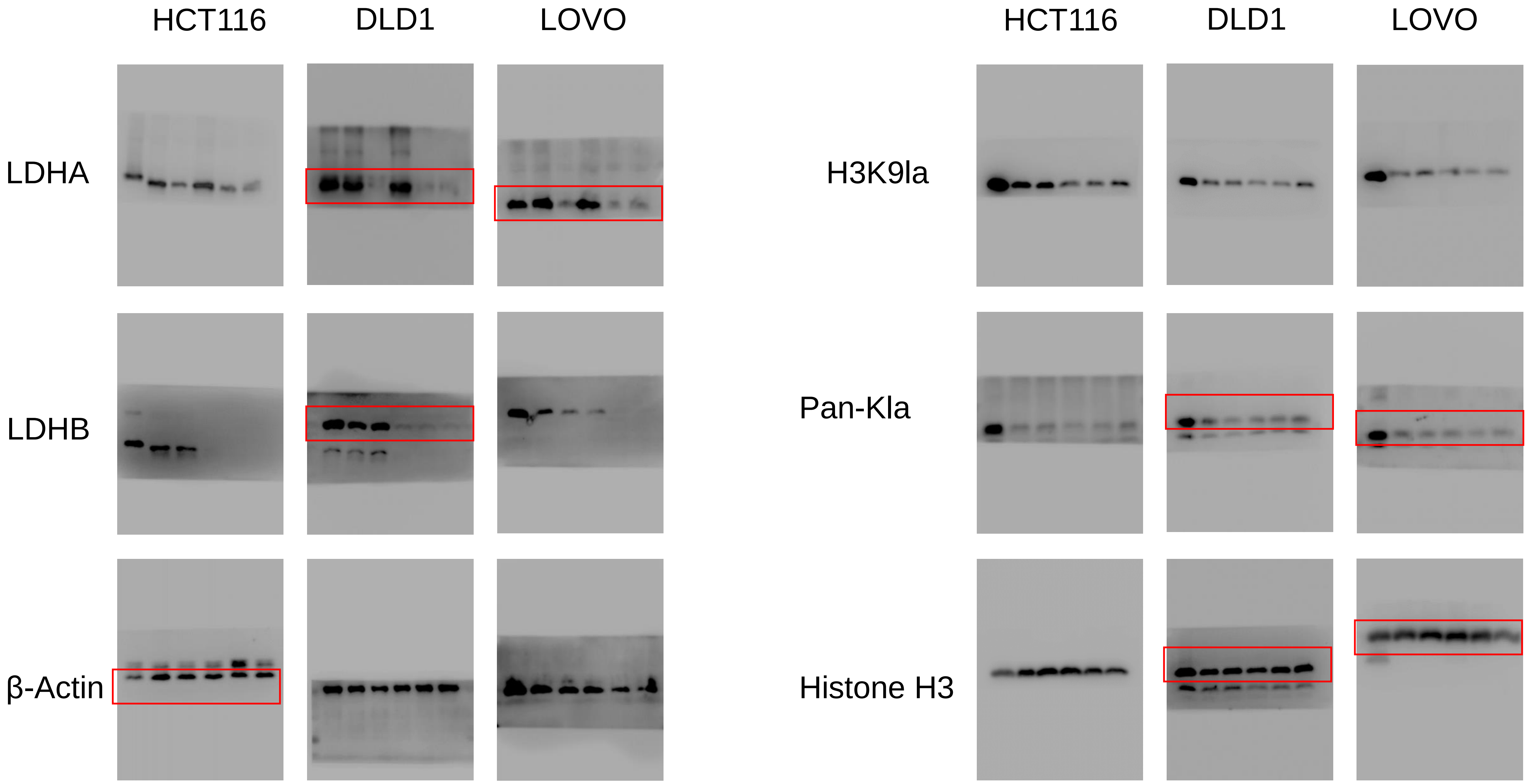

HCT116
DLD1
LOVO
LDHA
LDHB
β-Actin
HCT116
DLD1
LOVO
H3K9la
Pan-Kla
Histone H3

Figure 3 G

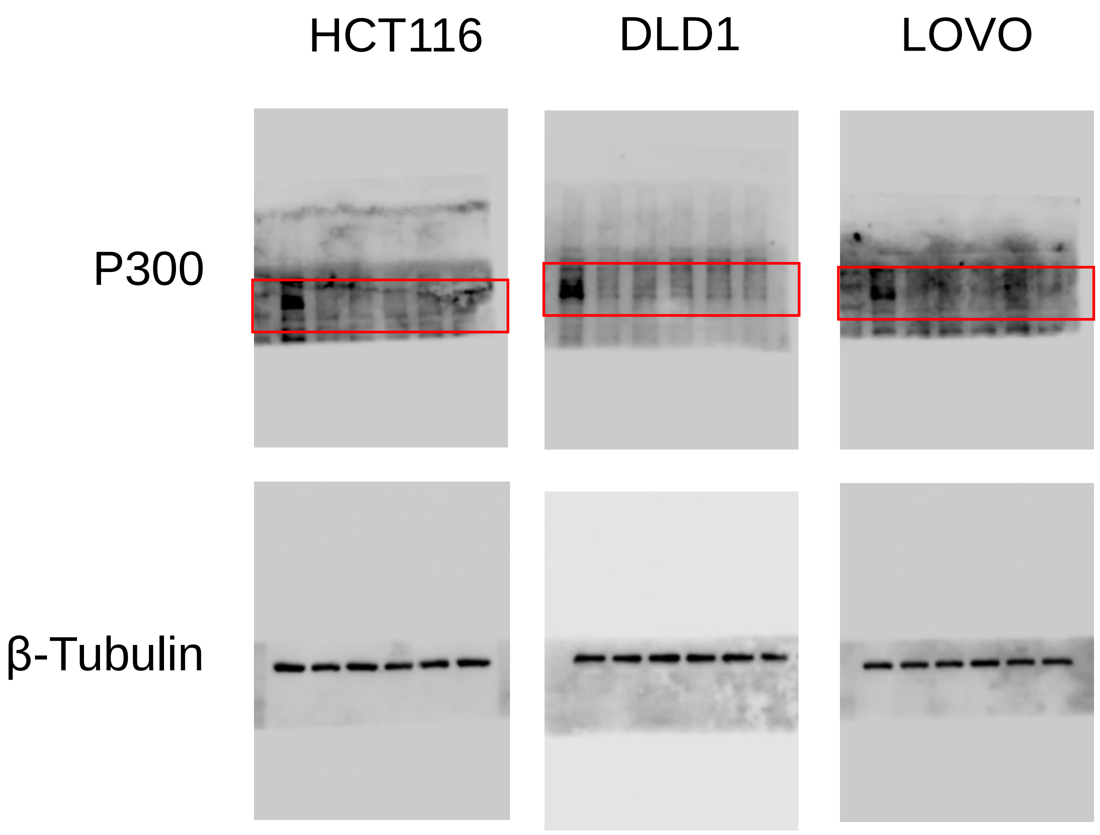

Figure S1 F

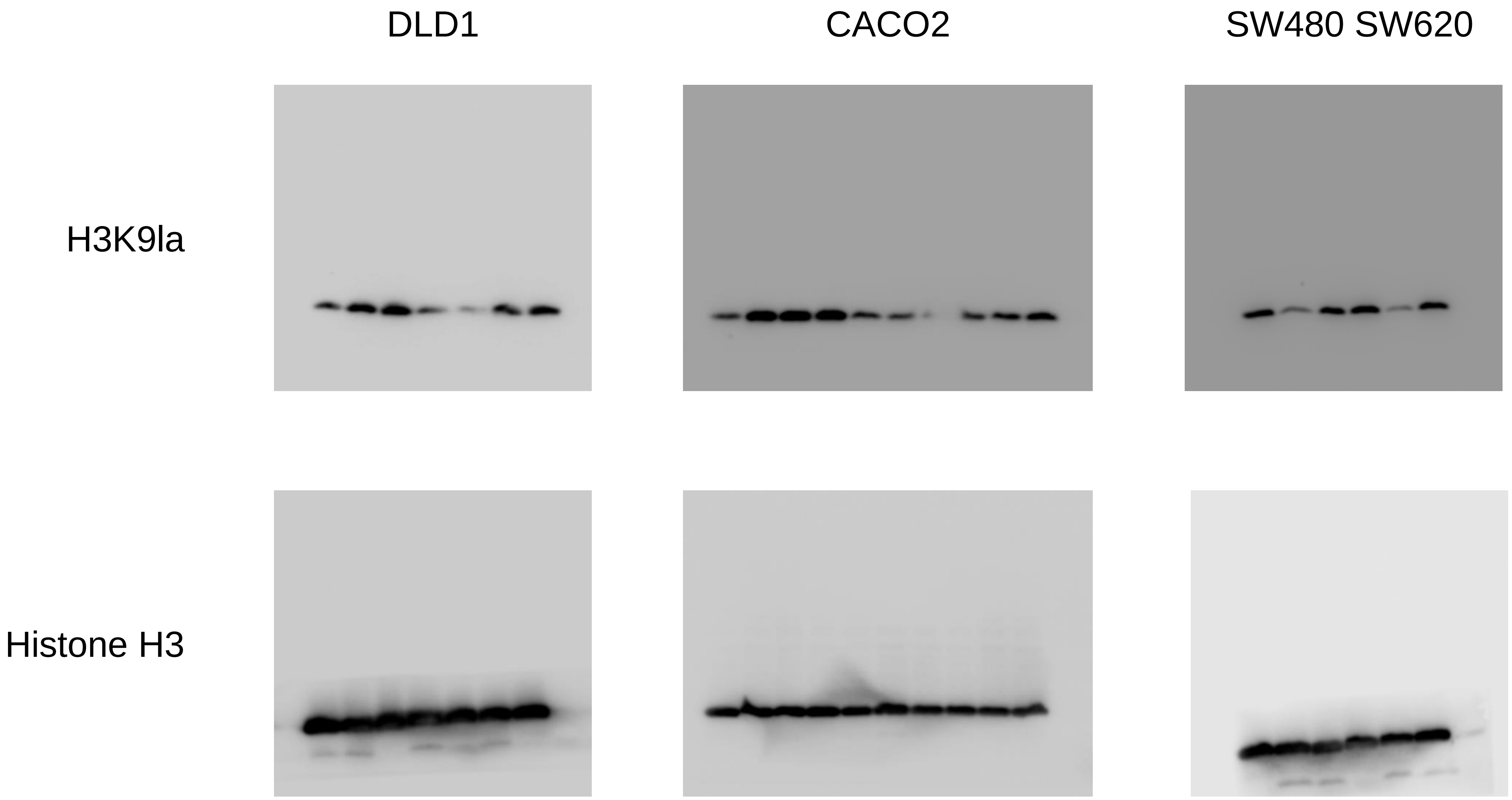

Figure S8 C

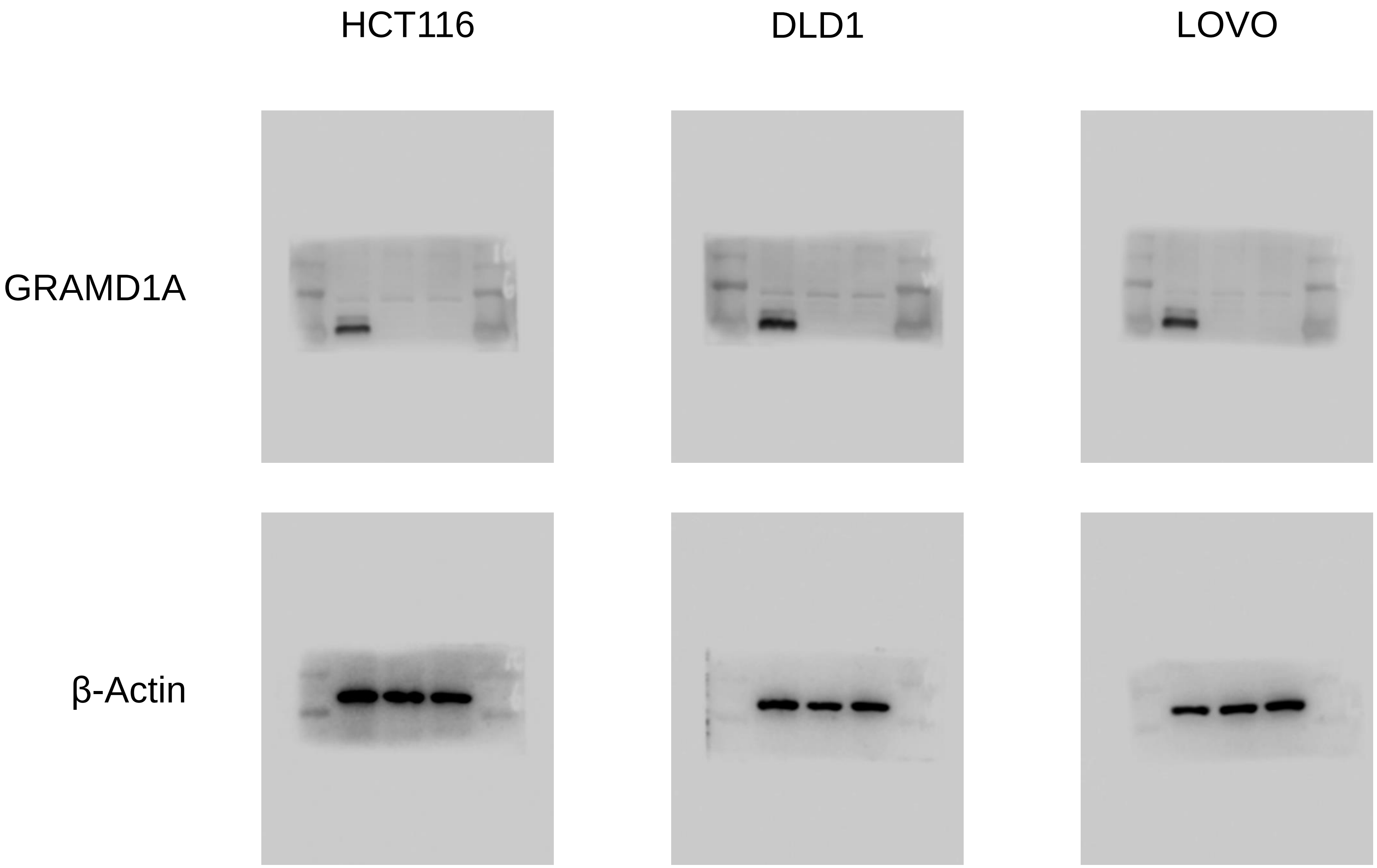

Figure S8 D

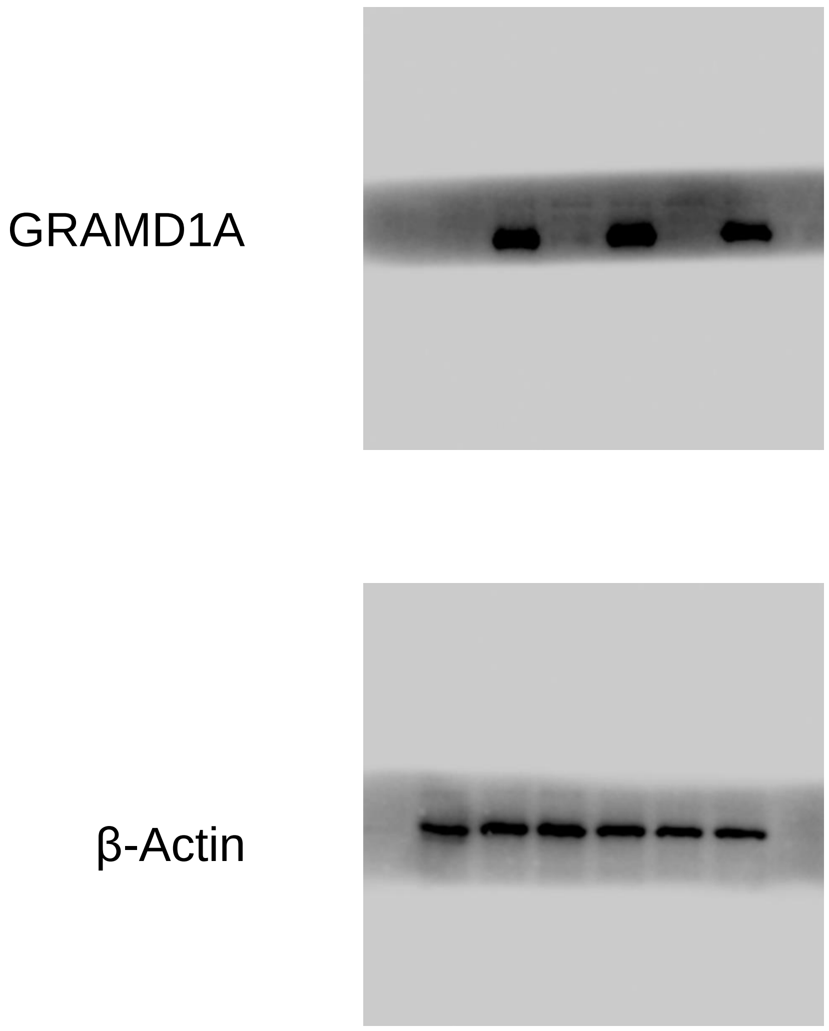

Figure S11 B

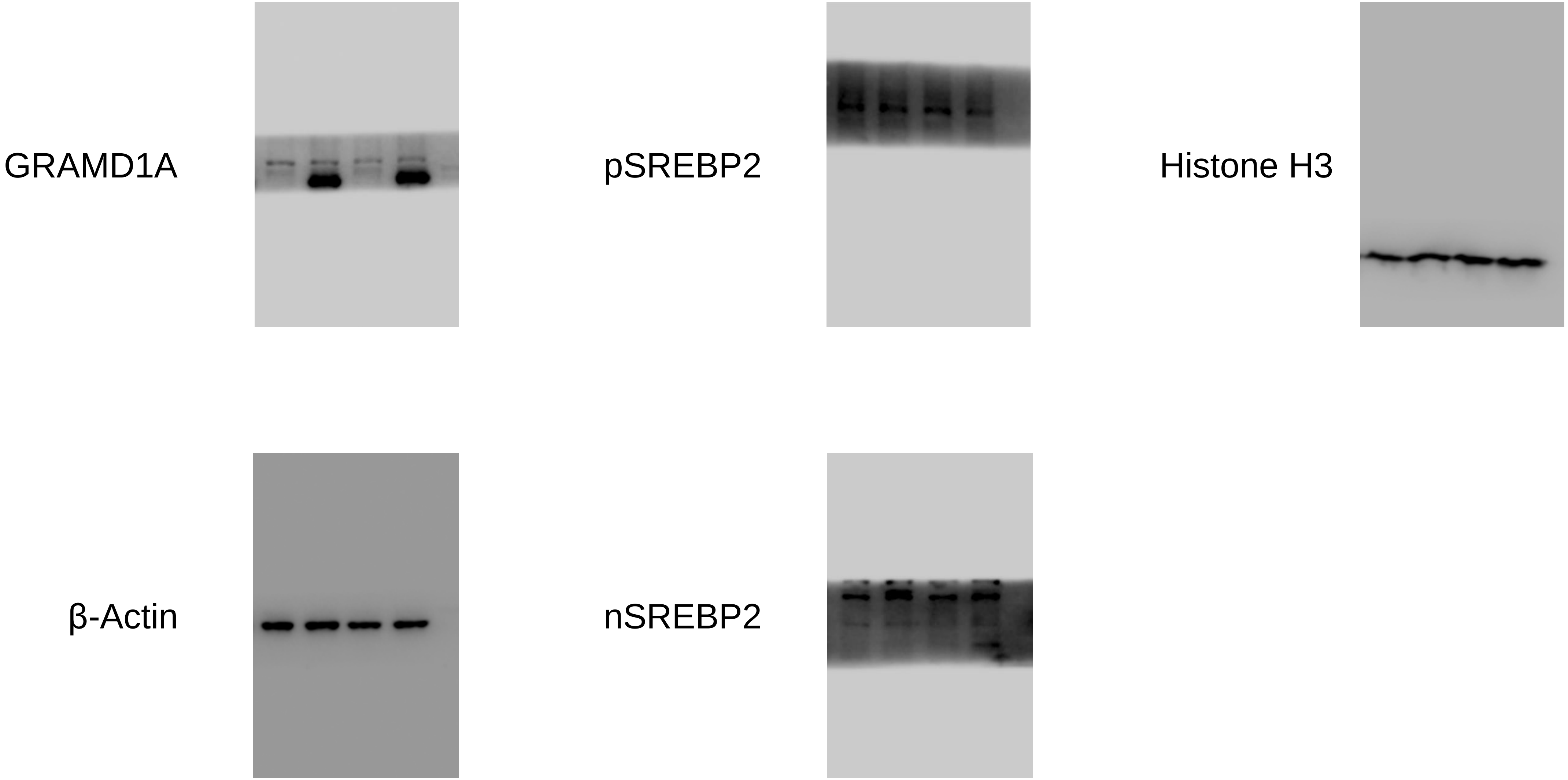

Supplement: Supplementary file 2 — Supplement Material-uncropped western blots [file 41418_2025_1533_MOESM2_ESM.pdf]
